# NICU sensory experiences associated with positive outcomes: an integrative review of evidence from 2015–2020

**DOI:** 10.1038/s41372-023-01655-y

**Published:** 2023-04-07

**Authors:** Roberta Pineda, Polly Kellner, Rebecca Guth, Audrey Gronemeyer, Joan Smith

**Affiliations:** 1 Chan Division of Occupational Science and Occupational Therapy, University of Southern California, Los Angeles, CA USA; 2 Keck School of Medicine, Department of Pediatrics, Los Angeles, CA USA; 3 Program in Occupational Therapy, Washington University School of Medicine, St. Louis, MO USA; 4 Center for Clinical Excellence, BJC HealthCare, St. Louis, MO USA; 5 Department of Quality, Safety, and Practice Excellence, St. Louis Children’s Hospital, St. Louis, MO USA

**Keywords:** Paediatrics, Rehabilitation

## Abstract

To inform changes to the Supporting and Enhancing NICU Sensory Experiences (SENSE) program, studies investigating sensory-based interventions in the NICU with preterm infants born ≤32 weeks were identified. Studies published between October 2015 to December 2020, and with outcomes related to infant development or parent well-being, were included in this integrative review. The systematic search used databases including MEDLINE, Cumulative Index to Nursing and Allied Health Literature, the Cochrane Library, and Google Scholar. Fifty-seven articles (15 tactile, 9 auditory, 5 visual, 1 gustatory/olfactory, 5 kinesthetic, and 22 multimodal) were identified. The majority of the sensory interventions that were identified within the articles were reported in a previous integrative review (1995–2015) and already included in the SENSE program. New evidence has led to refinements of the SENSE program, notably the addition of position changes across postmenstrual age (PMA) and visual tracking starting at 34 weeks PMA.

## Background

The neonatal intensive care unit environment (NICU) has been described as noisy and chaotic, and high-risk infants in the NICU are often exposed to invasive and painful medical interventions [1] and lack positive and consistent forms of sensory exposures [2]. The provision of consistent forms of positive and appropriately-timed sensory exposures to high-risk infants in the NICU can improve the safety and quality of care in the NICU. This is important, as very preterm infants require care in the NICU for an average of 3 months after birth [3], and the adverse NICU environment can have negative effects on early brain structure and function [1, 3]. Early brain development is susceptible to external stimuli [4, 5], and this modifiable factor could improve not only the NICU experience for parents and infants, but lead to better infant and parent outcomes.

In 2015, an integrative review was conducted to identify positive sensory exposures for high-risk infants in the NICU. The review identified 88 articles [6] with evidence over the previous 20 years (1995–2015). A large portion of literature supported the use of kangaroo care (also termed skin-to-skin), music and language exposure, and multimodal interventions starting at 25–28 weeks postmenstrual age (PMA). Such interventions have evidence supporting improvements in infant development, sleep, and physiology, as well as lower maternal stress. Although evidence exists that sensory interventions (kangaroo care, massage, music) relate to better parent and infant outcomes [7–10], most interventions for preterm infants in the NICU were implemented inconsistently and/or for short periods of time across studies [6], reducing their impact.

The initial integrative review was the first step in the development of the Supporting and Enhancing NICU Sensory Experiences (SENSE) program. The SENSE program includes specific doses and targeted timing of interventions such as massage, auditory exposure, rocking, holding, and kangaroo care [11]. The goal is to empower parents to engage in the described sensory activities with their infant(s). In addition to the initial integrative review [6], a rigorous process of protocol development for the SENSE program took place, which included expert input from a multidisciplinary group of 108 health care professionals that defined sensory interventions implemented across different NICUs, 3 multidisciplinary focus groups that provided a critical review of the guideline [12, 13], and interviews with 20 mothers of preterm infants who gave input on feasibility of implementing the SENSE program in the NICU [14]. All of these results were integrated to develop the evidence-based SENSE program.

Since then, a pilot study of 30 preterm infants who received the SENSE program compared to 50 historical controls demonstrated the feasibility of implementing the SENSE program in a level IV NICU and preliminary evidence of a positive impact on parent confidence and infant neurobehavior [15]. A randomized clinical trial of 70 parent-infant dyads identified more lethargy among infants who received the SENSE program, even after controlling for medical and social factors [16]. Better language outcomes on the Ages and Stages Questionnaire at 1 year of age were observed in the infants who received the SENSE program, but this was no longer significant after controlling for medical and social factors. Implementation research has identified that the SENSE program can be adopted with good fidelity [17]. An additional study examining the impact of the SENSE program on infants (*n* = 110), parents, and health care professionals reported that NICU personnel identified that having bedside information on appropriate sensory exposures (included in the SENSE program) enhanced their ability to deliver care and information to the caregiver [18]. Parents who received the SENSE program reported feeling better prepared for the transition to home and very satisfied with the quality of care they received in the NICU. Improved feeding outcomes in the group that received the SENSE program were also reported, with infants in the SENSE group demonstrating a decrease in the number of days between first gavage feeding and full oral feeding.

The SENSE program was made available to other hospitals in June of 2018, and more than 400 hospitals around the world have obtained the SENSE program. The cost of the SENSE program is used to support distribution, with no direct financial benefit to an entity or individual. NICU practice continues to evolve, as does the evidence that supports NICU care. Therefore, it is important to update the SENSE program as new evidence becomes available. Here, we report on an updated integrative review of evidence from 2015–2020 related to sensory-based interventions in the NICU to inform changes made to the SENSE program in 2022.

## Methods

### Purpose

The purpose of this review is to report evidence from October 2015–December 2020 related to sensory-based interventions in the NICU associated with positive infant and parent outcomes, in order to inform refinements to the SENSE program.

### Procedures

An integrative review was used to highlight the most relevant evidence related to sensory exposures in the NICU from a range of clinical research methodologies. Various study designs (systematic reviews, randomized controlled trials, quasi-experimental with subjects assigned to groups without use of randomization, crossover, or single-group repeated measure studies) published over a 5-year period (2015–2020) were considered for inclusion. The population of interest was preterm infants born ≤32 weeks gestation who had a sensory-based intervention that commenced prior to 36 weeks PMA while in the NICU. Studies that imposed a quantifiable environmental sensory exposure (tactile, auditory, vestibular, kinesthetic, visual, olfactory/gustatory, or multimodal) during the NICU stay were included. The comparison group received either (1) no identified sensory intervention, (2) standard of care, (3) varying levels of the same or similar intervention, or (4) a different sensory exposure. Interventions could be performed by healthcare workers, research team members, or parents. Relevant outcomes included infant behavioral outcomes (such as fewer observations of stress), neurobehavioral outcomes (including higher scores on standardized neurobehavioral or neurodevelopmental testing), parent well-being (less reported stress, anxiety, depression on standardized measures), and other parental outcomes (better confidence, more reports of engagement with infant in the NICU). Studies with outcomes related solely to pain or feeding were excluded, as these complex constructs deserve their own investigation and programming. Samples of healthy infants were excluded, as this review intended to define sensory exposures for medically complex preterm infants in the NICU. Studies with sample size <30 and without an a priori calculation of power that was met were excluded. Due to their inclusion in the previous integrative review (and related to the heterogeneity of outcome measures across studies), we included outcomes of bone health, growth, gastrointestinal function, lab values, infant physiology (fewer desaturation events, heart rate, respiratory rate), and length of stay in this review. However, we have denoted those studies as ones that will not inform refinements to the SENSE program, because they are a departure from the primary outcome of interest. The primary outcome of interest was infant neurobehavior or neurodevelopment.

See Table 1 for the exclusion criteria for this review. See Table 2 for search criteria and keywords.

**Table 1:** Study exclusion criteria.

Population
Populations with mean or median EGA greater than 32 weeks
Populations with mean or median postmenstrual age greater than 36 weeks at time of intervention
Populations with a purposeful sample of healthy infants (defined as 3 or more of the following factors: never on oxygen, never on medications, no intraventricular hemorrhage or other perinatal brain injury, Apgar scores >7 at 1 or 5 min, or never had sepsis)
Interventions
Interventions aimed at reduction of external stimuli (e.g., headphones to reduce noise)
Interventions aimed at reducing acute pain (e.g., during heel stick or endotracheal suctioning)
Breastfeeding interventions
Therapeutic touch that includes non-touch or energy-balancing techniques
Pacifier-activated sound (includes use of a learning element)
Vibrating pacifiers (includes use of a learning element)
Breathing bear (no direct intervention to the infant)
NIDCAP (interventions individualized for each infant rather than a uniform intervention)
Interventions specifically for pain or feeding that are not sensory interventions
Non-relevant outcomes
Outcomes not related to infant development or parent well-being
Incidence of diagnoses such as brain injury, retinopathy of prematurity, patent ductus arteriosus, or necrotizing enterocolitis
Breastfeeding measures or feeding outcomes
Study design and other factors
Studies published before 2015 or after 2020
Studies with a sample size <30 without an a priori power calculation or sample size not attained
Studies with unclear or incomplete methods, statistical analysis, or results
Pilot or feasibility studies (unless power calculation was done and met sample size requirements)
Studies without a comparison group (case reports or case series)
Systematic reviews that included studies with different EGA and PMA criteria
Primary studies included as part of a relevant systematic review
Studies printed in languages other than English
Studies not published in a peer-reviewed journal (conference abstracts or dissertations)

*EGA* gestational age, *NIDCAP* Newborn Individualized Developmental Care and Assessment Program, *PMA* postmenstrual age.

A systematic search for studies published from October 2015 to October 2020 was performed using databases including MEDLINE (via PubMed), CINAHL (Cumulative Index to Nursing and Allied Health Literature), the Cochrane Library, and Google Scholar. Reference lists of included studies were also searched.

We have included studies that report outcomes on physiology, bone health, gastrointestinal function, lab values, growth, and length of stay but have not considered these studies when refining the SENSE program due to their departure from the main outcome of interest, infant neurobehavior or neurodevelopment.

**Table 2:** Search criteria, keywords, and sample search strategy.

Subject	Keywords	MeSH Terms
Auditory	music therapy, music, Bach, Mozart, lullaby, singing, auditory stimulation, adult talk, parent talk, maternal AND heartbeat OR voice OR speech OR sound	Music Therapy; Singing; Acoustic Stimulation; Voice; Speech
Gustatory/Olfactory	colostrum, oral immune therapy, sucrose, flavor, gustatory, taste, oral hygiene, oral care, buccal care, smell, scent, olfactory, odor	Colostrum; Sucrose; Taste Perception; Taste; Flavoring Agents; Oral Hygiene; Smell; Olfactory Perception; Odors
Kinesthetic	kinesthetic, range of motion, physical therapy, exercise, physical activity, physiotherapy, passive limb movement, extension AND flexion	Kinesthetic; Range of Motion; Articular; Musculoskeletal Manipulations; Motor Activity; Physical Therapy Modalities; Movement; Exercise
Tactile	tactile stimulation, touch, tactile, massage, skin contact, skin-to-skin, kangaroo care, kangaroo mother care, acupressure, facilitated tuck, containment, hand hugs, holding	Touch; Tactile stimulation; M technique; TAC TIC; Massage
Vestibular	rock, bounce, swing, hammock, vestibular	Vestibule; Labyrinth; Motion; Proprioception
Vision	Eye contact, eye engagement, visual contact, visual engagement, eye-to-eye, mobile, light AND cycled OR exposure OR dim OR reduction, visual AND stimulus OR toy OR intervention OR novelty OR pattern	Photic Stimulation; Pattern Recognition; Visual; Color Perception; Lighting; Light
Multimodal	multimodal, multiple sensory, ATVV	
**combined with**		
Infant	infant, newborn, neonate, preterm, premature, low birthweight, LBW, VLBW, ELBW	Infant; Premature; Infant, Low Birth Weight

Sample Search Strategy		
Population	1 Infant OR infant* OR newborn*[tiab] OR neonat*[tiab] 2 preterm*[tiab] OR pre-term*[tiab] OR prematur*[tiab] OR “low birthweight”[tiab] OR “low birth weight”[tiab] OR lbw[tiab] OR vlbw[tiab] 3 1 AND 2 4 Infant, Premature 5 Infant, Low Birth Weight 6 #3 OR #4 OR #5	
Interventions	7 Music therapy OR music*[tiab] OR Bach[tiab] OR Mozart[tiab] OR lullab*[tiab] OR Singing OR singing[tiab] 8 Mothers OR mother*[tiab] OR maternal[tiab] 9 Voice OR voice[tiab] 10 Speech OR speech[tiab] 11 “sound simulation”[tiab] 12 #9 OR #10 OR #11 13 #8 AND #12 14 Acoustic Stimulation 15 “auditory stimulation”[tiab] 16 heartbeat*[tiab] OR “heart beat”[tiab] 17 “adult talk”[tiab] OR “parent talk”[tiab] 18 #7 OR #13 OR #14 OR #15 OR #16 OR #17	
Combined	19 #6 AND #18	

Searches of individual sensory categories also generated multimodal studies for inclusion.

### Search strategy

A systematic search for studies published from October 2015 to October 2020 was performed using databases including MEDLINE (via PubMed), CINAHL (Cumulative Index to Nursing and Allied Health Literature), the Cochrane Library, and Google Scholar. Reference lists of included studies were also searched for relevant literature. Searches were performed separately for each sensory topic (tactile, auditory, visual, kinesthetic, vestibular, olfactory/gustatory, and multimodal). A combination of search terms was used, including those focused on preterm infants and the sensory exposure(s) of interest.

### Study screening

**Fig. 1:**
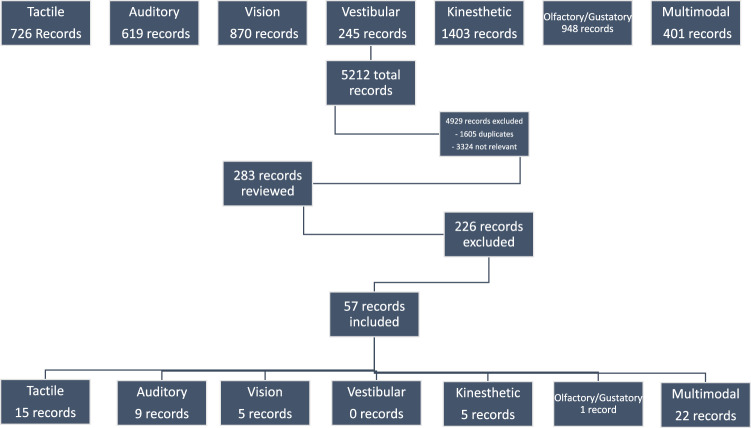
Flow diagram of studies identified during this review. The total number of articles identified are listed followed by how many were excluded for different reasons, followed by how many articles were reviewed across each sensory system.

Two reviewers set the search engine and screened articles for inclusion. One reviewer (AG or RG) first screened studies for inclusion by title. In situations where the title was unclear, the abstract was retrieved for review. The full text articles of potentially relevant studies were reviewed for final inclusion by both reviewers. If relevance of an intervention or inclusion of a study was unclear after review by both reviewers, it was resolved through discussion with the review team (RP, AG, RG, PK, and JS). See Fig. 1 for the flow diagram defining the total number of articles reviewed and how many remained after exclusions.

### Data extraction

One reviewer performed data extraction that was checked for accuracy by a second reviewer (AG or RG). Extracted information included study design, sample size, country of origin, intervention (including frequency, duration, timing), estimated gestational age (EGA) at birth, PMA at intervention, study inclusion/exclusion criteria, and study outcomes and results. When results from the same sample were reported in multiple publications, they were reported together in this review as a single study. When it was unclear if samples came from the same cohort, authors were contacted for confirmation.

### Study quality

Assessment of study quality was independently performed by two reviewers (AG and RG), and disagreements regarding study quality were resolved by discussion among the two reviewers until consensus was achieved. Systematic reviews were assessed for methodological quality using the Documentation and Appraisal Review Tool (DART) [19]. The remaining studies were assessed for quality using a modified version of a tool developed by the United Kingdom’s National Institute for Health and Care Excellence (NICE [20]). The tool evaluates studies for selection bias (randomization, allocation concealment, group comparability at baseline), performance bias (groups received the same care, blinding of participants and healthcare workers), attrition bias (equal follow-up time, completion of treatment, complete outcome data), detection bias (appropriate length of follow-up, precise definition of outcomes, valid and reliable outcomes, blinding of investigators or outcome assessor), and other bias (statistical methods, issues related to specific study designs). Each factor was rated as yes/adequate, no/inadequate, or unclear. Several of these factors were not relevant for single-group repeated measures studies (Appendix B).

## Results

**Fig. 2:**
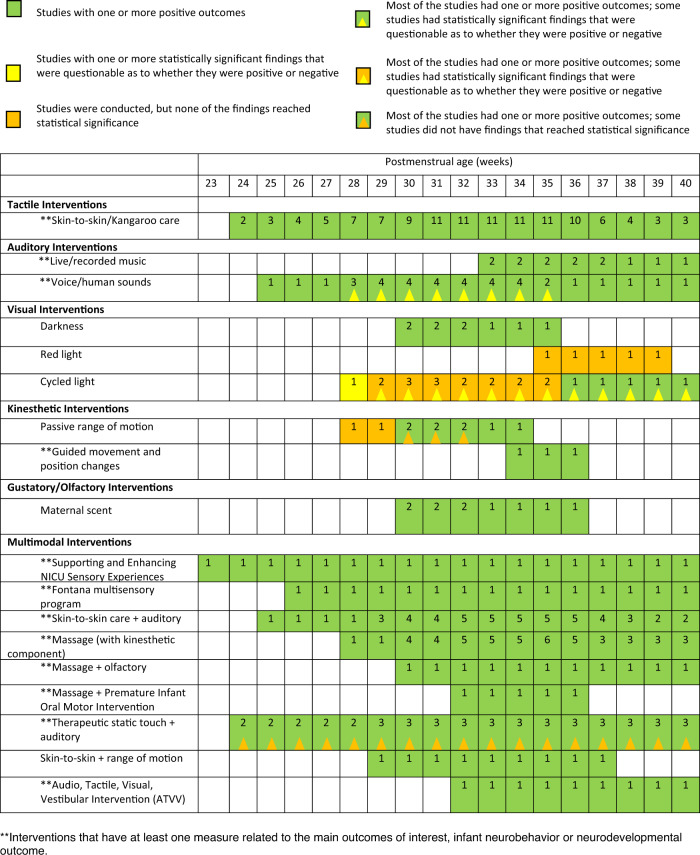
Different sensory interventions studied across postmenstrual age (PMA) from 2015–2020. The number in each box indicates how many studies were conducted with at least some of the sample receiving interventions at that particular PMA. Some studies did not list sufficient information to estimate PMA at time of intervention and, thus, were not included in this figure.

See Fig. 1 for a breakdown of the articles reviewed during the integrative review process. See Fig. 2 for evidence of the different types of interventions across PMA. See Appendix A for the 49 cohorts (and 57 published articles) included in this review (13 cohorts on tactile across 15 publications, 6 cohorts on auditory across 9 publications, 3 cohorts on kinesthetic across 5 publications, 1 cohort on olfactory/gustatory across 1 publication, 5 cohorts on visual across 5 publications, and 21 cohorts on multimodal interventions across 22 publications).

### Synthesis of findings

Given the significant heterogeneity of studies and their outcomes, study findings could not be combined quantitatively, but were summarized qualitatively. Evidence related to each type of sensory intervention was defined across each PMA, to determine at what age of maturity evidence existed to support specific interventions (Fig. 2). Further, all outcomes were reported for each sensory exposure, but outcomes related to neurobehavior or neurodevelopmental outcomes were highlighted. See Appendix C for interventions across PMA from 1995–2020, inclusive of evidence from previous and current integrative reviews.

### Tactile

The previous 1995–2015 review identified evidence-based tactile exposures to be gentle human touch [21–24], massage [25–27], and kangaroo care or skin-to-skin [7, 28–49]. This new 2015–2020 integrative review added 15 articles (13 cohorts) all of which described kangaroo care being done for 15 min [50] to 2 h [51, 52] starting as early as 24 weeks PMA [53]. Two studies evaluated kangaroo care specifically in the delivery room immediately after birth, starting at 25 weeks PMA [54, 55]. The frequency of kangaroo care across studies varied and ranged from 1 time per day [50] to an average of 4 times per day [56, 57]. The length of time that kangaroo care was studied ranged from 1 day [53, 58–60] to 3 weeks [50]. No new tactile interventions were identified since the previous review. However, multimodal interventions often included a tactile component (see “Multimodal” section below). Positive outcomes associated with increased duration and/or frequency of kangaroo care included stabilized respiratory rate [58, 61], improved maternal-infant attachment [54, 55, 61, 62], decreased parental stress [50, 54, 55, 61, 63], lower heart rate [58, 60], increased short-term electromyographic activity of the biceps brachii and hamstrings [59], increased oxygen saturation [51], decreased infant salivary cortisol levels [64], increased salivary oxytocin levels for infants and parents [64], decreased anxiety for parents [64], decreased risk of early postpartum depression or impaired bonding [54, 55], increased weight gain, fewer episodes of apnea, decreased use of formula, improved sleep, and decreased crying [52]. One study related a tactile exposure to the primary outcome of interest (neurobehavioral or neurodevelopmental outcome): longer duration of kangaroo care (120 versus 60 min over 7 days) starting at 31 weeks PMA was related to better scores for attention, arousal, regulation, non-optimal reflexes, quality of movement, handling, excitability, and lethargy on the NICU Network Neurobehavioral Scale [51].

### Auditory

The previous 1995–2015 review identified evidence-based auditory exposures to be live music/singing [65, 66], recorded music/singing/maternal voice [67–71], and recorded maternal biological sounds [72, 73]. This new 2015–2020 integrative review added an additional 9 articles, representing 6 different cohorts, on auditory interventions. Auditory interventions described included recorded music or voice and live music or voice. Auditory interventions started as early as 25 weeks PMA [74] and lasted from 8 min [75–78] to 30 min 5x/day for a total of 150 min per day [74]. Studies occurred for as short as 1 day [79, 80] and as long as 6–10 weeks [74]. Some studies used headphones, and others did not. Live musical instruments used included the pentatonic harp [80]. Recordings included Brahm’s lullaby, punji, bells, harp, and voice (folkloric lullaby, humming, singing, reading, talking). Auditory interventions were also described within other bundled multisensory programs [15, 81]. Not all studies reported decibel levels; those that did reported ranges between 65–75 decibels [74, 82], while some packaged programs, such as the SENSE program, described auditory interventions at the American Academy of Pediatrics’ recommendation of ≤45 decibels [83]. One study identified use of an auditory stimulus played 5–15 decibels above background noise [79]. Studies over the past 5 years identified positive outcomes associated with recorded maternal lullaby from 28–34 weeks PMA, recorded music from 33–40 weeks PMA, recorded female voice from 25–40 weeks PMA, and live harp music from 33–37 weeks PMA. In the multimodal category, additional studies were identified that combined auditory stimulation (namely maternal voice and music) with kangaroo care, therapeutic touch, and massage. An auditory component (voice/singing with daily dose recommendations based on infant PMA) was also included as part of the packaged SENSE program [15]. Outcomes related to recorded music starting at 33 weeks PMA included increased brain connectivity [76, 78] and improved white matter maturation and larger amygdala volumes [77]. No differences in neurodevelopmental outcome were noted in groups that received recorded music as an intervention [75, 79]. Recorded maternal voice starting as early as 28 weeks (talking, reading, and/or singing) was related to parents having less fear, discontent with baby, burden, and increased rates of breastfeeding as well as increased infant oxygenation and decreased heart rate [84]. One study related auditory exposures (30 min of recorded maternal voice, 5 times per day for a total of 150 min per day) starting 2 weeks after birth (as early as 25 weeks PMA) over 6–10 weeks to the primary outcome of interest: recorded maternal voice was related to improved neurodevelopmental outcome at 5 months on the Griffith Scales (but not at 20 months) [74].

### Visual

The previous 1995–2015 review identified cycled light as the only evidence-based visual intervention [85–87]. This new 2015–2020 integrative review added an additional 5 articles, all of which examined cycled light with an attempt to better pinpoint reactions to light as well as appropriate timing of initiation of cycled light. In these studies, cycled lighting was introduced as early as 28 weeks PMA [88, 89], however, the comparator was starting cycling at 36 weeks which did not enable pinpointing whether timing of cycling at 28 weeks compared to 32 weeks PMA (when cycling is introduced in most instances) differed. Lux levels ranged from 1–30 lux at night and 40–600 lux during the day, with most falling around 200–250 lux during the day. There was also a new Cochrane review of cycled light published in 2017 [90], but all of the articles described were already captured in our previous integrative review. Four articles with visual components were included in the multimodal category as visual stimulation was a component of other sensory programs or experiences, either via cycled lighting [15], with eye contact during music therapy (20-min sessions, 2–3x/week) [81], with a darkened environment using a cover over the incubator until initiation of oral feeding [91], or with visual tracking [92]. Visual tracking of a parent’s face or toy was described starting at 34 weeks PMA 6 times per week with positive outcomes related to improved visual performance [92]. Further, one study investigated the use of red light, but there were no significant outcomes related to its use [93]. There were no studies that related visual exposures alone to the primary outcome of interest.

### Kinesthetic

The previous 1995–2015 review identified evidence-based kinesthetic interventions to be physical therapy [94, 95] with most outcomes related to bone health. This new 2015–2020 integrative review added an additional 5 articles, representing 3 cohorts. Most reported on passive range of motion (ROM) with bone-related outcomes. None of the studies on passive ROM alone demonstrated significant differences in the outcomes of interest. Movement exercises started as early as 28 weeks PMA [96] and consisted of ~7–10 min of exercise each day, with some occurring 2 times per day. Three articles (all reporting on the same cohort) assessed guided movement which consisted of different positions to improve postural control, head control, and midline orientation. This was done for 10 min twice per day at 34, 35, and 36 weeks PMA, with positive outcomes on the Test of Infant Motor Performance (TIMP) identified [97–99]. One study with a kinesthetic component was categorized as multimodal, with passive ROM coupled with gentle joint compression during kangaroo care between 29–37 weeks PMA, with positive outcomes identified and listed in the multimodal section below [51]. Another multimodal sensory program, the SENSE program, included a 2-min movement opportunity up to 1–8 times per day, increasing in frequency dependent upon the infant’s PMA and tolerance [15]. Six studies, categorized as multimodal, used the Field protocol for infant massage, which consists of 5 min of tactile input, 5 min of vestibular input, and an additional 5 min of tactile input for a total of 15 min of intervention [100–105]. One of these studies found positive outcomes, including increased weight gain, associated with conducting Field massage three times per day for 15 consecutive days, between 30–35 weeks PMA, with the infant in kangaroo care [100]. A similar protocol consisted of a 15-min massage with tactile and vestibular components and was implemented 2 times per day for 14 days between 30–36 weeks PMA [106]. Although not directly related to the outcomes of interest, daily range of motion exercises were associated with higher tibial speed of sound measures as an indicator of bone mineral density and specifically osteopenia [96]. Two studies (in one cohort) found kinesthetic interventions to be related to the primary outcome of interest: parent-administered guided movement starting at 34 weeks PMA (10 min twice per day for 3 weeks) to improve postural control, head control, and midline orientation was associated with higher *z*-scores on the TIMP at 37 weeks PMA [98, 99].

### Gustatory and olfactory

The previous 1995–2015 review identified evidence-based olfactory/gustatory interventions as oropharyngeal colostrum, breast milk odor, or mother’s scent [107–109]. This new 2015–2020 integrative review added one additional article on gustatory/olfactory interventions which described a parent scented positioning device which was used between 30–36 weeks PMA for at least 12 h at a time [110]. Another study in the multimodal category compared maternal voice to breast milk odor to incubator cover (darkened environment) to standard of care and found that the breast milk odor group had the shortest duration to full oral feeding [91]. Infants in the breast milk odor group were exposed one time per day for 3 h at a time to 5 ml of breast milk poured into a sterile sponge and positioned 5 centimeters from the infant between 30–32 weeks PMA. Additionally, a study in the multimodal category looked at massage with coconut oil, which reportedly has a subtle odor [111]. Positive outcomes were identified for infants who received massage with 5 ml of coconut oil three times per day between 30–40 weeks PMA and included higher mean weight gain, less hypothermia and apnea, better Neonatal Skin Condition Scores, and better scores on the Developmental Assessment Scale for Indian Infants at 3, 6, and 12 months. There were no studies that related unimodal olfactory/gustatory exposures to the primary outcome of interest.

### Multimodal

The previous 1995–2015 review identified evidence-based multimodal interventions as Auditory, Tactile, Visual, and Vestibular (ATVV), Family Nurture Intervention, Hospital to Home Transition-Optimizing Premature Infant’s Environment (H-Hope), massage with aromatic oil, kangaroo care coupled with auditory exposure, and massage intervention with a kinesthetic component [8, 106, 112–146]. This new 2015–2020 integrative review added an additional 22 articles (representing 21 cohorts) on multimodal interventions. Multimodal interventions described included the SENSE program, the Fontana multisensory program, kangaroo care coupled with an auditory intervention, massage (with a kinesthetic component), massage coupled with olfactory input, massage coupled with the Premature Infant Oral Motor Intervention (PIOMI), therapeutic static touch coupled with auditory input, kangaroo care coupled with passive ROM, and ATVV. Tactile and kinesthetic stimulation was related to increased neuromuscular maturity [101], improved sleep [102], increased weight and length [103, 104], increased head circumference [104], and decreased heart rate [103]. Kangaroo care and kinesthetic stimulation was related to improved weight gain [100]. Kangaroo care with music was related to improved mother-infant attachment [147], decreased maternal anxiety [148], and improved weight gain [148]. Massage was associated with increased length, larger chest circumference, increased number of bowel movements, and decreased frequency of pre-feed gastric residual [106]. Massage with oil was associated with decreased weight loss, decreased incidence of hypothermia, decreased incidence of apnea, better skin condition, and decreased length of hospitalization [105, 111]. Touch and auditory stimulation were associated with improved sleep and more stable oxygenation [149]. ROM with compression was associated with higher serum phosphorus levels, lower levels of urinary calcium/phosphate, higher bone mineral density, increased weight gain, and lower alkaline phosphate levels [51]. In relation to the outcomes of interest, bundled multimodal sensory interventions were associated with improved visual reception, improved early learning composite scores, and improved infant neurobehavior [15, 81] as well as improved maternal confidence [15]. Massage with coconut oil was related to improved motor and mental developmental quotients on the Development Assessment Scale for Indian Infants [111], and PIOMI with massage was related to improved neurodevelopmental outcomes on the Ages and Stages Questionnaire [150].

### Vestibular

The previous and new reviews added no new studies pertaining to isolated vestibular interventions, though several vestibular interventions were coupled with other sensory interventions as noted in the other sections.

## Discussion

The key findings of this review are that (1) there is additional evidence that further supports previously reported sensory-based interventions, (2) there are a few additional sensory-based interventions described in the literature not reported in the previous integrative review, (3) there continue to be significant differences in the reporting of sensory exposure outcomes, dosages, and timing, making it challenging to combine studies or have a cohesive understanding of sensory exposures across PMA, and (4) many studies fail to clearly identify the PMA at which the interventions were conducted. Understanding evidence that emerged from 2015–2020 has led to refinements to the SENSE program. The evidence from 2015–2020 informed changes to the SENSE-II program, including the addition of kinesthetic interventions (position changes) and the use of visual tracking.

Kangaroo care, or skin-to-skin care, appears to have the strongest evidence among sensory-based interventions in the NICU setting. Additional support of kangaroo care, including the use of it starting in the delivery room, emerged in this new review. Further, auditory interventions were defined with one study estimated as starting as early as 25 weeks PMA [74]. Consistent with previous reports, the use of live music or recorded music is not well-reported until at least 32 weeks PMA. Evidence to support timing of cycled light prior to 32 weeks was not isolated, however, evidence to avoid cycled lighting during earlier PMA also does not exist. There continues to be significant variability in cycled light protocols, with variation in lux levels used during day/night cycles. Further, the use of visual tracking of a parent face or toy starting at 34 weeks PMA was a new addition to the evidence [92], as previously there was very little to support visual interventions except within the context of the light environment. Guided movement in different positions was also a new intervention described in the literature starting at 34 weeks PMA [97–99]. Evidence on olfactory interventions continue to support parent smell and breast milk with some description of multimodal interventions that included the smell of coconut oil during massage. Multiple new bundled interventions have also emerged in the literature.

There remains little evidence investigating long-term outcomes from sensory interventions. Kangaroo care was related to less infant distress, more comfort, better attachment, improved parent mental health, improved infant sleep, decreased infant crying, and improved infant neurobehavior at term [50–52, 54–58, 61–64]. Auditory interventions were related to brain structure on MRI, development at 5 months of age, as well as personal-social, emotional stability, and language outcomes [75–78]. Guided movement related to improvements on neurobehavioral outcome measures at 3 months of age [99]. Multimodal interventions also were related to long-term neurodevelopmental outcomes [111, 150]. However, a large amount of the current literature remains focused on shorter term outcomes that have relevance that is questionable for the purposes of this review. An absence of evidence does not mean these interventions do not improve long-term outcomes. Rather, it is a call for research to better understand the long-term impact of sensory-based interventions in the NICU.

It is important to note that research of interventions in a tightly controlled study is different from implementation of interventions in the real world, with the latter often times lacking highly skilled personnel, being done during other concurrent interventions, and without use of strict exclusion criteria [151]. Careful consideration as to whether each intervention can be done for most infants at a given PMA is complex, and vulnerability of infants in the real-world context must be carefully evaluated. Subsequently, adaptations may be needed [152] in order to adequately document the effects of interventions following implementation.

### Limitations of included studies

There is a possibility of publication bias, where only studies reporting positive outcomes were published and included in this review. In addition, most studies included multiple outcome measures, many of which did not reach significance. We reported studies that were included in this review in Fig. 2, color coded depending on if the articles had at least one positive outcome that reached significance; however, studies with multiple outcomes (especially those with findings that did not reach significance) may not be well-represented in the PMA tables. We included multiple research designs in an effort to capture all appropriate literature related to improving the sensory environment, and it is possible that lower quality, non-randomized designs could have biased or decreased the strength of the review findings. Of the studies that were randomized, many did not specify methods clearly or report allocation concealment. These, in addition to incomplete or weak assessments of participants at baseline, placed many of these studies at high-risk for selection bias. While participants could not be blinded, and it may be difficult to blind parents and healthcare workers to the intervention, few studies attempted to blind the outcomes assessor. Completeness of treatment and follow-up was also difficult to ascertain, as studies infrequently reported the number of infants by group with complete outcomes data and reasons for loss to follow-up. Most interventions were short in duration and were not conducted across the majority of hospitalization, which limits the strength and generalizability of findings. Finally, generalizability is limited due to the integration of multiple different studies conducted on different populations and in different environments.

### Limitations of this review

This review was limited by its focus on parent outcomes and infant neurodevelopmental outcomes. Therefore, evidence with outcomes related to pain, breastfeeding success, nutritional/growth, feeding outcomes, and other important clinical markers may have been excluded. Some studies that reported on the aforementioned outcomes are reported in this review when multiple outcomes were reported, including the outcomes of interest. This review did not include studies printed in languages other than English and did not include non-published literature. Only 1 reviewer screened studies and performed initial data extraction. Exclusion of studies with a sample size less than 30 may have excluded relevant literature. The size and scope of this review also did not allow us to comprehensively, consistently, and repeatedly follow-up with individual study authors in situations where methods or data were missing or unclear. In addition, this review is limited by lack of common interventions and outcomes, as well as failure to elucidate the PMA at which interventions were administered, making it difficult to combine results into a cohesive whole. Because of the significant heterogeneity of the studies included, interpretation is largely qualitative.

## Conclusion

In conclusion, this review informed changes to the SENSE program. These additions included refinements of language, expansion of acceptable light protocols prior to 32 weeks PMA, and the addition of position changes, movement, and visual tracking activities. While this new evidence from 2015–2020 informed proposed changes to the SENSE program, suggested changes were then vetted through an expert advisory team. Since the basis of the SENSE program is already established, and research on its efficacy is ongoing, future reviews that will take place every 5 years may focus on randomized clinical trials as the basis for change and only include studies that clearly define the timing (PMA) of the intervention and have outcomes related to the primary outcome of interest, neurobehavioral or neurodevelopmental outcome. Using such a systematic approach of study design inclusion will decrease the challenges associated with inclusion of studies with different study quality and enable the value to be placed on rigorous study designs to inform further change to the SENSE program. Committing to a review of new studies every 5 years will ensure the SENSE program remains updated, based on current evidence.

## Supplementary information

Appendix A

Appendix B

Appendix C

## Data Availability

Data available within the article or its Supplementary Materials.
