# Supplementary material for: NICU sensory experiences associated with positive outcomes: an integrative review of evidence from 2015–2020
Source: J Perinatol. 2023 Apr 7;43(7):837–48. doi: 10.1038/s41372-023-01655-y (PMC10325947; doi:10.1038/s41372-023-01655-y)
Supplement: Supplementary file 1 — Appendix A [file 41372_2023_1655_MOESM1_ESM.pdf]

Appendix A

1. Table of tactile sensory studies

**Author, Year, Country**	**N**	**Study Design**	**Intervention and Dose (frequency, length, timing)**	**Age (GA at birth, PMA at intervention)**	**Outcomes**	**Results**
Cho 2016<br><br>Korea	40	Non-randomized Trial	2 groups: Kangaroo Care vs no kangaroo care<br><br>Intervention: 3x/week, between 2:30-4, 30-min duration, total of 10 times	Mean GA:30.12 week (KC)<br><br>28.81 weeks (control)<br><br>Corrected age: 33.7 weeks (KC)<br><br>33.0 weeks (control)<br><br>Estimated time of intervention approx. 33 weeks PMA to term	Maternal-infant attachment, maternal stress, RR, oxygen saturation, temperature, weight	**RR:** RR after kangaroo care significantly differed between groups (p=0.20). RR of preterm babies in experimental group (54 to 45) was significantly stabilized compared with control group (46 to 47).<br><br>There were no significant differences in body temperature and oxygen saturation.<br><br>**Maternal-Infant attachment**: significantly changed at post-test, the KC group had higher maternal-infant attachment scores than control group (p<0.001) (4.48 to 4.74; control 4.24 to 4.48).<br><br>**Maternal stress:** significantly differed between groups with KC being related to decreased maternal stress (4.67 to 3.76; vs control 4.49 to 4.40)
Ozdel 2018 Turkey	30	Single-group repeated measure	2 groups: Infants fed in KC position vs prone position<br><br>Intervention: infants were fed in KC position for 30 min at a time, outcomes were recorded 3 hours after<br><br>Prone: infants were fed in prone position for 30 min	GA: 28-36 weeks<br><br>Mean PMA: 30.2 ±2.63 weeks	Gastric residual volume, vital signs, and comfort, distress, pain	**Comfort score**: 30 min after feeding, KC group had lower comfort scores (6.7±1.68) compared to prone position (9.50±3.73, p<0.001)). 3 hours after feeding, mean comfort scores were lower in KC group (9.83±4.05) than prone group (18.00±5.76, p<0.000)<br><br>**Distress score**: Mean distress scores were lower in KC group than prone group both 30 min (0.10±0.30 vs 1.06±1.11, p<0.000) and 3 hours after feeding (1.10±1.15 vs 4.20±1.86, p<0.000)<br><br>**Heart rate**: 3hrs After feeding, heart rate was lower in KC group (147.6 bpm) compared to

Author, Year, Country	N	Study Design	Intervention and Dose<br><br>(frequency, length, timing)	Age (GA at birth, PMA at intervention)	Outcomes	Results
						prone position (156.6 bpm, $p<0.001$). no significant difference 30 min after feeding.<br><br>**RR**: 30min and 3 hours after feeding KC group had slightly lower respiratory rate compared to prone group (55.03±5.86 vs 58.56±6.73, $p<0.007$; and 55.556±5.17 vs 58.76±6.68, $p<0.011$, respectively)<br><br>**SpO_2**: mean SpO_2 values were slightly lower 30 min after feeding for KC group (95.96±2.47 vs 97.43±2.59, $p<0.019$) but were not significantly different 3 hours after feeding<br><br>**Body temperature**: no significant differences in body temperature<br><br>Gastric residual volume: No significant difference in gastric residual volume<br><br>Pain: no significant difference in pain scores
Kurt 2020	60	Non-randomized trial	2 groups: Kangaroo care vs control<br><br>Intervention: infant wearing only a diaper and hat was placed on mother in prone and horizontal position on bare chest, covered with preheated cover. Sessions were 30 min, twice a day, for 5 days.<br><br>Control: infants visited by parents once a day, fed them, made hand contact	GA: <37 weeks<br><br>Mean GA<br>32.77±2.65 (KC) 32.97±2.47 (control)<br><br>Chronological age: unclear	Maternal attachment (Maternal attachment scale score; MAS)	**Maternal attachment:** Mean MAS of kangaroo group (35.03±5.54) was significantly higher than control group (29.87±4.66, $p<0.001$)

Author, Year, Country	N	Study Design	Intervention and Dose (frequency, length, timing)	Age (GA at birth, PMA at intervention)	Outcomes	Results
Coşkun 2019<br>Turkey	80	Randomized Controlled trial	2 groups: kangaroo care vs standard care<br>Intervention: 1x/day, 15-20min, for 5 days a week, 3 weeks total	GA: 27-36 weeks<br>Mean GA: Unclear mean EGA (age broken down in categories)<br>Chronological age: ≤28 days<br>Approximate time of intervention was 31-40 weeks	Stress levels (Parental Stressor Scale score), amount of milk production	**Stress Levels:** Mothers in the KC group were reported to have improved Parental stressor scale scores in NICU subdimension and total scale average scores from pretest to posttest, had lower posttest scores compared to mothers in the standard care group (Images and sounds subdimension 1.92 vs 2.71, p<0.000; Infant's appearance and behaviors sub-dimension 1.83±0.71 vs 2.76,±0.65 p<0.002; Parental role sub-dimension 1.83±0.52 vs 2.95±0.57, p<0.000; Average total score 1.86±0.57 vs 2.81±0.49, p<0.000)
Diniz 2019<br>Brazil	47	Randomized Controlled Trial	2 groups: kangaroo position vs control<br>Intervention: use of band to support the newborn against the adult's thorax, in a prone and vertical position, keeping skin-to-skin contact for a duration of 2 hours.	GA: 28-37 weeks<br>Mean GA: 31.93 weeks (KC)<br>31.91 weeks (control)<br>Mean corrected age at time of evaluation 33.83 weeks (KC)<br>33.93 weeks (control)<br>Estimated time of intervention was approx. 27-37 weeks PMA	Muscle activity	**KC group**: There was a significant variation of electromyography activity values between the three recorded positioning moments for both the bicep brachii and hamstrings in the intervention group.<br>**Control:** There was no significant differences in electromyography activity values for the control group.<br>Authors concluded that kangaroo positioning increases short-term electromyographic activity of biceps brachii and hamstrings.
**El-Farrash 2019<br>Egypt	120	Randomized Controlled Trial	3 groups: Kangaroo care vs Kangaroo care long durations vs standard care<br>Intervention: infants wearing only a diaper were placed upright in a prone position on parents' chest, direct skin-to-skin contact, legs and arms flexed. Duration of KC was either 60 min or 120 min daily, 7 days (parents allowed to continue until discharge);	GA: 31-35 weeks<br>Mean GA: 32.5±1.02 (KC 60 min)<br>32.45±1.39 (KC 120 min) 32.35±1.04 (controls)<br>Intervention started within the first 24 hours after birth<br>Approximate PMA of intervention was 31-36 PMA	Salivary cortisol, temperature, respiration rate, heart rate, and oxygen saturation, and neurobehavioral performance (Neonatal Intensive Care Unit Network Neurobehavioral Scale)	**Neurobehavioral performance:** Both KC groups had higher scores for attention, arousal, regulation, nonoptimal reflexes, and quality of movements and lower scores for handling, excitability, and lethargy compared to control group (p<0.05).<br>**Oxygen saturation:** After first KC session, a greater improvement in mean O_2 saturation (+4.17±0.52 vs +3.48±0.84) was observed in the longer duration group compared to 60-min group (p<0.05)

Author, Year, Country	N	Study Design	Intervention and Dose<br><br>(frequency, length, timing)	Age (GA at birth, PMA at intervention)	Outcomes	Results
			conventional group-parents could hold for 15-30 minutes			**Temperature:** after first KC session, there was also a greater improvement in mean temperature (+0.61±0.11 vs +0.43±0.16) in the longer duration group compared to 60-min group
Vittner 2018<br><br>USA	28	Randomized cross-over	2 groups: Skin-to-skin contact for 1 session 60 min long vs control for a 3 day study period	PMA: 30-36 weeks<br><br>Mean GA: 33±1.57 weeks<br><br>Chronological age: 3-10 days	Infant and parent salivary oxytocin, salivary cortisol levels	**Salivary cortisol:** infant salivary cortisol levels decreased significantly (p<0.001) during SSC compared to before or after SSC. No significant difference for mother or father<br><br>**Salivary oxytocin**: salivary oxytocin levels increased significantly during SSC for mothers (p<0.001), fathers (p<0.002), and infants (p<0.002)<br><br>**Anxiety**: anxiety levels were significantly lower during SSC compared to before or after, for both mothers (p<0.001) and fathers (p=0.003)
Buil 2019 a<br><br>Buil 2019 b (same cohort)<br><br>France	34	Quasi-experimental	SSC + Supported Diagonal Flexion (SDF) positioning vs SSC + vertical positioning (control)<br><br>Only recorded the first 15 min of SSC<br><br>Once to several times daily (mean of 4 times a day), mean duration for vertical group was 77 min and 103 min for SDF group, for 15 consecutive days	27 to < 32 weeks GA<br><br>Mean GA 32.4 weeks	Mother-infant communication, infant communication behaviors, Infants' stat of consciousness (Assessment of Preterm Infants' Behavior scale), duration of infants' and mothers' smiles, gazed and vocalizations, temporal proximity in 1 sec window, risk of depression (Edinburgh Postpartum Depression Scale), stress (Parental Stressor Scale	**State of consciousness:** infants were in a state of drowsiness majority of the time (58% of time for SDF, 70% for vertical group). Both groups spent 15% of time in active sleep. SDF group spent 17% in deep sleep and vertical group 0%. 9% in quiet awake state for SDF vs 13% for vertical group.<br><br>**Vocal Production**: SDF group had slightly more recorded vocalizations per minute compared to vertical group (3.68 vs 1.06, p<0.0001), but vocalization duration did not differ between groups (401 ms vs 318 ms, p=0.32).<br><br>**Gazes at mother:** infants spent most of time with eyes closed (SDF 67% vs vertical 62%, p=-.95). time spent with eyes open did not differ (SDF 13% vs vertical 8.6%, p=0.46)

Author, Year, Country	N	Study Design	Intervention and Dose<br><br>(frequency, length, timing)	Age (GA at birth, PMA at intervention)	Outcomes	Results
					Neonatal Intensive Care Unit and Perinatal Post-traumatic stress disorder Questionnaire)	**Smiles:** no statistical analysis conducted. Only recorded 16 smiles from 6 infants in SDF group, and 21 smiles from 8 infants in vertical group.<br><br>**Mothers' behavior:** SDF mothers produces more vocalizations than vertical group (11.2 per min vs 5.7, p=0.022), but duration did not differ. SDF mothers gazed more frequently (3.4 per min vs 1.4, p=0.003, respectively), and for longer (64% of time vs 35%, p=0.010, respectively). SDF mothers spent more time smiling than vertical mothers (9.3% of time vs 2.0%, p=0.023)<br><br>**Temporal proximity**: infant target behaviors were temporally closer to mother target behaviors in the SDF group than vertical group for vocalizations (p=0.004), gazes (p=0.009) and smiles (p=0.003).<br><br>**Maternal Stress:** PSS:NICU scores after SSC were all lower in the SDF group except parental role alteration scores, but were not significantly different<br><br>**Maternal depression risk**: EPDS scores were similar for both groups prior to SSC (13.8 and 12.9), after SSC both scores fell under 10 but SDF group had a slightly larger decrease than vertical group (7.0 vs 9.0, respectively) though not significantly different.
Dongre, 2020, India	30	Single group repeated measures	KFC: 90 min KFC on 3 consecutive days	Included GA 28-35 weeks;<br><br>Mean EGA 30.53 ± 2.5; unclear age at study start	Parental stress scale: NICU (4 domains)	**Parental stress scale – total score (median, IQR):** lower after KFC (98.5, IQR 72-133.8) vs. before KFC (112.5, IQR 91.25-156.3), p=.006<br><br><u>Subscales (all values median, IQR)</u>

<table>
<tr><th>Author, Year, Country</th><th>N</th><th>Study Design</th><th>Intervention and Dose

(frequency, length, timing)</th><th>Age (GA at birth, PMA at intervention)</th><th>Outcomes</th><th>Results</th></tr>
<tr><td></td><td></td><td></td><td></td><td></td><td></td><td>Sights and sounds: no difference before KFC (11.5, IQR 7.75-16) vs. after KFC (10, IQR 7-13.25), p=.17

Baby looks and behaves: no difference before KFC (58, 38.5-73.5) vs. after KFC (57.5, 31.75-69.75), p=.052

Relationship with baby and parental role: no difference before KFC (24.5, 14.25-35) vs. after KFC (25, 15.5-41), p=.96

Staff behaviors and communication: lower after KFC (11, 3.5-26.25) vs. before KFC (18, 11.75-37.25), p=.001</td></tr>
<tr><td>*Forde, 2020,

USA</td><td>51</td><td>Randomized controlled trial</td><td>KMC: 1 hour of KMC on the Days 3 and 4 of life

Controls: Incubator care on Day 3 and 1 hour of KMC on Day 4 of life</td><td>Included GA 24-36 weeks;

Mean EGA: KMC 32.0 ± 2.6 weeks; Control 31.4 ± 2.1 weeks; Intervention started on Day 3 of life</td><td>Splanchnic oxygen saturation, abdominal temperature, perfusion index (all measured at 15 min intervals during the observation period), and urinary biomarkers (allantoin, uric acid, xanthine, and hypoxanthine; measured pre/post intervention each day)</td><td>Splanchnic tissue oxygen saturation: “measures were homogenous across the groups” (data were not reported in the study)

Abdominal temperature (°C) and Perfusion Index (%): not significantly different between groups at any time point (data reported graphically)

Urinary biomarkers (metabolite(uM)/Cr(uM)):
<table>
<tr><th></th><th>KMC

(mean±SD)</th><th>Control

(mean±SD)</th><th>p-values</th></tr>
<tr><td colspan="4">Uric Acid</td></tr>
<tr><td>Day 3-Pre</td><td>.422±.298</td><td>.398±.298</td><td rowspan="4">Group: 0.562;

Time: 0.025</td></tr>
<tr><td>Day 3-Post</td><td>.463±.300</td><td>.433±.300</td></tr>
<tr><td>Day 4-Pre</td><td>.418±.298</td><td>.364±.298</td></tr>
<tr><td>Day 4-Post</td><td>.332±.298</td><td>.284±.305</td></tr>
</table></td></tr>
</table>

Author, Year, Country	N	Study Design	Intervention and Dose<br><br>(frequency, length, timing)	Age (GA at birth, PMA at intervention)	Outcomes	Results			
						**Xanthine**			
						Day 3-Pre	.014±.011	.013±.011	Group: 0.193;<br><br>Time: 0.042
						Day 3-Post	.014±.011	.013±.011	
						Day 4-Pre	.021±.011	.016±.011	
						Day 4-Post	.016±.011	.014±.012	
						**Hypoxanthine**			
						Day 3-Pre	.017±.038	.010±.037	Group: 0.586;<br><br>Time: 0.645
						Day 3-Post	.015±.037	.009±.039	
						Day 4-Pre	.029±.038	.013±.040	
						Day 4-Post	.014±.039	.015±.041	
						**Allantoin**			
						Day 3-Pre	.191±.185	.244±.177	Group: 0.026;<br><br>Time:<br><br>0.072
						Day 3-Post	.273±.128	.295±.128	
						Day 4-Pre	.188±.137	.276±.131	
						Day 4-Post	.170±.134	.241±.128	
Mehler 2020 and Hucklenbruch-Rother, 2020,<br><br>Germany	88	Randomized Controlled Trials	2 groups: 60 min maternal skin-to-skin contact in the delivery room vs 5 min visual contact<br><br>*Although could be in multimodal section due to comparing a tactile intervention to a visual intervention, we put this study in	GA: 25-32 weeks<br><br>Mean GA: 29±2 weeks (SSC)<br><br>29±2 weeks (VC)<br><br>Intervention started 45 min following birth	Mehler 2019: Mother-child interaction (MCI), salivary cortisol, maternal depression, stress and bonding at 6 months;<br><br>Hucklenbruch-Rother 2020: expression of 6 stress-response genes	**Mother-child interaction:** SSC group showed higher quantity of maternal motoric (18 vs 15, p=0.030), and infant's vocal (7 vs 5, p=0.044) and motoric responses (20 vs 15, p=0.032). combined score of maternal and infant responsive behavior was higher for SSC group (86 vs 71, p=0.041)<br><br>**Maternal depression:** SSC group mothers had lower risk of bother early postpartum depression			

Author, Year, Country	N	Study Design	Intervention and Dose<br><br>(frequency, length, timing)	Age (GA at birth, PMA at intervention)	Outcomes	Results
			the tactile section as we assume the visual contact was a control		in peripheral white blood cells (from blood sample at hospital discharge)	(15% vs 45%, p=0.003) and impaired bonding (score 3 vs 5, p=0.031)<br><br>**Salivary cortisol:** lower levels of salivary cortisol after heel lance for VC group compared to SSC group (52%% vs 76%, p=0.071), but results were not statistically significant.<br><br>**Stress-response gene expression:** expression of 3 stress response genes (CRHR2 [p=.032], NR3C1 [p=.039], SLC6A4 [p=.011]) was significantly lower in the SSC group compared to the visual contact group. There were no differences between groups for expression of CRHR1, AVP, or HTR2A. Expression of CRHR2 was also associated with infant responses during mother-child interaction at 6 months.
Shattnawi, 2019, Jordan	89	Non-randomized Trial	2 groups: Short duration skin-to-skin contact vs controls<br><br>SSC: infant only wearing a diaper and head cap rested on mothers' bare chest, 60-120 min sessions per day, for 5 days	GA: 26 to <37 weeks<br><br>Mean GA: 32±2.48 weeks (SSC)<br><br>33±2.41 weeks (controls)<br><br>Intervention initiated within 12 hours postnatal age	Weight gain, feeding, sleeping, and crying patterns, as well as occurrence of apneas	**Weight:** compared to controls, SSC group had higher weight gain from day 3-5 (53.7 g vs 32.6 g, p<0.05)<br><br>**Apnea:** compared to controls, SSC experienced fewer number of apneas (48% vs 33.3%, p=0.001)<br><br>**Formula feeding**: SSC group was also less likely to use formula feeding (60% vs 90%), and more likely to use mixed feeding (formula and breastfeeding) at discharge (33.3% vs 10%)<br><br>**Sleep:** a higher proportion of infant in SSC group had good sleep all 5 days compared to controls<br><br>**Crying**: less infants in SSC group cried in a continuous patter over 5 days compared to controls

Author, Year, Country	N	Study Design	Intervention and Dose<br><br>(frequency, length, timing)	Age (GA at birth, PMA at intervention)	Outcomes	Results
*Sehgal, 2020,<br><br>Australia	40	Single group repeated measures	Single group intervention of SSC; assessments made 2 hours before SSC care and after 60 min of SSC while infant was still with the parent	Included GA 28-36 weeks; mean EGA 30.5±0.6 weeks; mean postnatal age at study 12 days (IQR 9-25)	Temperature (axillary), HR, RV stroke volume, RV fractional area change, TAPSE, TPV/RVETc, LV stroke volume, fractional shortening, SVC VTI, middle cerebral artery resistive index	**Temperature (axillary, mean±SD):** No difference before SSC (36.7±.07 °C) vs. after SSC (36.9±.07 °C, p=.07)<br><br>**HR (mean±SD):** Higher before SSC (154±2 bpm) vs. after SSC (146±2 bpm, p<.001)<br><br>**RV stroke volume (mean±SD):** Lower before SSC (1.26±.04 mL/kg) vs. after SSC (1.41±.05 mL/kg, p<.001)<br><br>**RV fractional area change (mean±SD):** Lower before SSC (26.5±.3%) vs. after (27.8±.4%, p<.001)<br><br>**TAPSE (mean±SD):** Lower before SSC (0.73±.03 cm) vs. after SSC (0.77±.03 cm, p=.02)<br><br>**TPV/RVETc (mean±SD):** Lower before SSC (0.27±.007) vs. after SSC (0.3±.008, p<.001)<br><br>**LV stroke volume (mean±SD):** Lower before SSC (1.24±.04 mL/kg) vs. after SSC (1.37±.04 mL/kg, p=.0001)<br><br>**Fractional shortening (mean±SD):** No difference before SSC (27.8±.1%) vs. after SSC (29.2±.1, p=.054)<br><br>**SVC VTI (mean±SD):** Lower before SSC (8.3±.3 cm) vs. after SSC (9.3±.3 cm, p<.001)<br><br>**Middle cerebral artery resistive index (mean±SD):** Higher before SSC (0.81±.02) vs. after SSC (0.74±.02, p=.0001)

Abbreviations: EGA, estimated gestational age; FiO_2, fraction of inspired oxygen; GA, gestational age; HR, heart rate; IQR, interquartile range; KC, kangaroo care; KFC, kangaroo father care; KMC, kangaroo mother care; MAS, maternal attachment score; PMA, postmenstrual age; RR, respiratory rate; RV, right ventricular; SpO_2, oxygen saturation; SSC, skin-to-skin contact; SVC, superior vena cava; TAPSE, tricuspid plane systolic excursion; TPV/RVETc, time to peak velocity to RV ejection time; VTI velocity time integral.

2. Table of auditory sensory studies

Author, Year	N	Study Design	Intervention Dose (frequency, length, timing)	Mean EGA of sample<br>Estimated PMA at intervention start	Outcomes	Results
*Jabraeili<br>2016,<br>Iran	66 total;<br>21 maternal lullaby,<br>25 Brahm's lullaby;<br>20 controls	Randomized controlled trial	Maternal lullaby: 15 min taped recording of mother reciting a folkloric lullaby at played at 65-70dB; Brahm's lullaby: 15-min of Brahm's lullaby (65dB); Controls: "routine auditory stimulation"; sound exposure delivered on 3 consecutive days for 15 min between 1000 and 1900	Mean EGA 31.45 to 32.16;<br>intervention started between 3 and 14 days after birth<br>Timing of intervention was at approximately 29-34 weeks PMA	SpO_2	**SpO_2** between groups was not different between groups immediately before (p=.63) and immediately after the intervention (p=.08) but was significantly different 20 minutes after the intervention (Brahm's lullaby 94.92±2.15 vs. maternal lullaby 93.70±2.71 vs. control 92.33±4.23, p=.02). When sessions across the 3 day period were compared, no differences between groups were detected during the first and second sessions; during the third session, SpO_2 in the Brahm's lullaby group was significantly higher than controls (mean difference 2.58, p=.02)
**Lejeune 2019a,<br>Lordier 2019a, Lordier 2019b,<br>Sa de Almeida 2020,<br>Switzerland<br>*These studies used the same population (confirmed by authors via email 8/12/20)	20 music;<br>19 controls (fewer were available at follow-up and included in each study)<br>* This study also included full term infants that did not receive an intervention but were used as a	Randomized controlled trial	Music group listened to 8 minutes of music (bells, harp, punji) 5 days a week via headphones; control group wore headphones for the same time/frequency without music	Mean EGA varied by study and patients who were available for follow-up, but ranged from 28 to 29 weeks; intervention started at 33 weeks PMA; lasted until approx. term	Lordier 2019a: fMRI at term equivalent age;<br>Lordier 2019b: fMRI (obtained at term equivalent age; music stimuli were played in 5 conditions, including music played at a faster tempo);<br>Lejeune 2019: BSID-III at 12 & 24 mos, Lab-TAB at 12 & 24 mos, Effortful Control Battery at 24 mos;<br>Sa de Almeida 2020: brain structure maturation at term equivalent age (measured by MRI)	**fMRI (Lordier 2019a):** "Preterm infants exposed to music […] have significantly increased coupling between brain networks previously showed to be decreased in premature infants: the salience network with superior frontal, auditory, and sensorimotor networks, and the salience network with the thalamus and precuneus networks."<br>**fMRI (Lordier 2019b):** "Auditory cortex functional connectivity with cerebral regions known the be implicated in tempo and familiarity processing were identified only for preterm infants with music training in the NICU. Increased connectivity between auditory cortices and thalamus and dorsal striatum may not only reflect their sensitivity to the known music and the processing of its tempo as familiar, but these results are also compatible with

Author, Year	N	Study Design	Intervention Dose (frequency, length, timing)	Mean EGA of sample<br><br>Estimated PMA at intervention start	Outcomes	Results
	comparison group in some analyses					the hypothesis that the previously listened music induces a more arousing and pleasant state."<br><br>**BSID-III:** no significant group effect was observed at 12 mos or 24 mos<br><br>**Lab-TAB:** there was no significant effect joy, anger, or fear reactivity between the preterm groups; some tests showed differences between full term and preterm infants with "less important" differences between preterm infants in the music group when compared to full term infants than between preterm controls and full term infants<br><br>**Effortful Control Battery:** no significant group effect was observed during any trial<br><br>**Brain structure maturation:** Music group infants had "significantly improved white matter maturation" and "larger amygdala volumes" compared to control group infants<br><br>*Because some findings were qualitative, they were difficult to summarize (e.g. fMRI, brain structure maturation)
Lejeune 2019b,<br><br>Switzerland	24 voice at<br><br>5 decibels above background noise (mean 68.7 + 5.7 decibels; 24 voice at 15 decibels above background noise (mean	Randomized controlled trial	Recorded female voice reading a 22-second text in French that ran in a loop and was played at +5 or +15 decibels above background noise	Mean EGA 30+3 for silence group and 29+6 for voice group; age at intervention<br><br>28-35 weeks PMA	Habituation with holding an object (smooth wooden cylinder or sharply angled prism) as measured by holding time, object discrimination in 2 test trials (conducted after habituation occurred by presenting the infant with a familiar or new-shaped object)	<u>+5 vs. +15 decibel voice groups</u><br><br>**Habituation (failures):** no difference between +5 decibel (25%) vs. +15 decibel (29.2%, p=.75)<br><br>**Total object holding time (mean, sec):** no difference between +5 decibel (133±83) vs. +15 decibel (142±109, p=.64)<br><br>**Discrimination:** infants in the +15 decibel group held the objects longer during the last 2 habituation trials (6.1 vs. 4.2 sec, p=.020) and two test

Author, Year	N	Study Design	Intervention Dose<br><br>(frequency, length, timing)	Mean EGA of sample<br><br>Estimated PMA at intervention start	Outcomes	Results
	69.8+5.2 decibels) ; 26 controls					trials (19.5 vs. 14.7 sec, p=.044) compared to the +5 decibel group<br><br>Voice (+5 and +15 decibel) vs. control group<br><br>**Habituation (failures):** more failures in the voice groups (27.1%) vs. controls (7.7%, p=.048)<br><br>**Total object holding time (mean, sec):** no significant difference (voice 137±95 vs control 106±64, p=.09)<br><br>**Trials to achieve habituation (mean):** more required in voice groups (6.1±2.1) vs controls (5.3±1.2, p=.05)
**Nöcker-Ribaupierre<br><br>2015,<br><br>Germany	48 total; 24 auditory stimulation;<br><br>24 controls	Randomized controlled trial	30 minutes of recorded maternal voice (free talking, reading, and/or singing and humming) placed 20cm from the infant's ear in the incubator played 5 times per day at 65-75 decibel for 150 minutes per day for 6-10 weeks	Mean EGA 28.5 (both groups);<br><br>Intervention started the second week after birth or when stable and continued to 36 weeks PMA; intervention occurred at approximately 25-40 weeks PMA	Griffith scales at 5 and 20 months; Columbia Mental Maturity Scales, a picture vocabulary test to assess verbal intelligence, logopedic test of understanding at 56 months; Heidelberg Test of Speech Development at 75 months (6 years); maternal report of milestones of development, maternal emotional stability (via interview); growth parameters (weight, length, head circumference)	**Griffith Scales – 5 months and 20 months**<br><br>Overall development: improved in maternal voice group at 5 mon (95±15 vs. 80±20, p=.007); not different at 20 mon (maternal voice 83±25 vs. control 78±23, p=.424)<br><br>Motor: not different at 5 or 20 mon<br><br>Personal-social: improved in maternal voice group at 5 mon (98±18 vs. 85±20, p=.019); not different at 20 mon (maternal voice 80±25 vs. control 78±23, p=.839)<br><br>Hearing and speaking: hearing not different at 5 mon; hearing/speaking not different at 20 mon<br><br>Vision-hand: improved in maternal voice group at 5 mon (97±21 vs. 78±26, p=.009); not different at 20 mon (voice 83±25 vs. control 79±25, p=.561)

Author, Year	N	Study Design	Intervention Dose (frequency, length, timing)	Mean EGA of sample<br><br>Estimated PMA at intervention start	Outcomes	Results
						Efficiency: improved in maternal voice group at 5 mon (89±21 vs. 71±24, p=.008); not different at 20 mon (voice 86±27 vs. control 78±30, p=.328)<br><br>**Milestones (mean, weeks after term)**<br><br>Fixation: not different (16±4 vs. 19±5, p=.069);<br><br>Smiling: not different (18±3 vs. 18±5, p=.639);<br><br>Babble: not different (21±4 vs. 24±6, p=.115);<br><br>Says "mama": not different (60±12 vs. 62±14, p=.580); 2-word sentences: faster for maternal voice group (71±14 vs. 83±16, p=.008); Sitting for 30 sec: faster for maternal voice group (40±10 vs. 49±11, p=.006); Crawling: not different (48±12 vs. 50±12, p=.571); Walking 5 steps: not different (68±14 vs. 76±15, p=.065)<br><br>**Heidelberg Language Development Test (mean):** only understanding of grammar structure was higher in the maternal voice group (51.72±11.20 vs. 43.11±15.02, p=.052); plural-singular, correction of grammar inconsistency, and sentences were not different between groups<br><br>**Maternal emotional stability:** maternal voice group mothers reported less fear (4 vs. 37%), less discontent with the baby (0 vs. 21%), less general burden of the family (0 vs. 25%), and more breastfeeding (50% vs. 12.5%)<br><br>**Measurements:** Body weight, body length, and head circumference not different between groups at hospital discharge or at follow-up<br><br>**Neurologist findings**: At 5 months, 8 (33%) maternal voice and 4 (17%) controls exhibited

<table>
<tr><th>Author, Year</th><th>N</th><th>Study Design</th><th>Intervention Dose

(frequency, length, timing)</th><th>Mean EGA of sample

Estimated PMA at intervention start</th><th>Outcomes</th><th>Results</th></tr>
<tr><td></td><td></td><td></td><td></td><td></td><td></td><td>moderate motor abnormalities, and 2 (8%) maternal voice and 7 (29%) controls exhibited severe motor abnormalities; 3 (12.5%) maternal voice and 8 (33%) controls exhibited moderate mental abnormalities, and 1 (4%) infant from each group had severe mental abnormalities. At 20 months, 3 (12.5%) infants from each group had moderate motor abnormalities, and 5 (21%) maternal voice and 10 (42%) controls had severe motor abnormalities; 5 (21%) maternal voice and 7 (29%) controls had moderate mental abnormalities, and 6 (25%) maternal voice and 8 (33%) controls had severe mental abnormalities.

*This had many findings– some are narratively summarized rather than with numerical values; some were omitted</td></tr>
<tr><td>Ranger

2018,

Germany</td><td>21 (had a power analysis and met the sample size estimate)</td><td>Randomized crossover</td><td>On a single day, each neonate received 2 sequential interventions that each lasted 2.25 hours, including one hour pre- and post-phases. The middle 15 minutes consisted of music or a control (no music) period. Music was live pentatonic harp with 2-min fade-in of arpeggios and ending with 2-min fade out of arpeggios; free melodies played in between in an adagio triple meter.</td><td>Mean EGA 32 ± 1 weeks; intervention at 2 ± 6 weeks; mean age at measurement 35 (+1). Intervention at approximately 33-37 weeks PMA</td><td>Desaturation events (SpO_2<90%), bradycardia events (HR<80), perfusion index, pulse transit time, heart rate variability measures (RMSSD, SDNN, pNN_{50}), maternal anxiety (German version STAI-X1), maternal rating of their child's emotional states before and after the intervention</td><td>
<table>
<tr><th></th><th>Music

Median (IQR)</th><th>Control

Median (IQR)</th></tr>
<tr><td colspan="3">Number of desaturation events</td></tr>
<tr><td>Pre-phase</td><td>2.0 (0, 5.0)</td><td>1.0 (0, 8.0)</td></tr>
<tr><td>Intervent.</td><td>0 (0, 1.8)</td><td>0 (0, 3.0)</td></tr>
<tr><td>Post-phase</td><td>2.5 (0, 8.5)</td><td>3.0 (0, 6.0)</td></tr>
<tr><td colspan="3">Number of bradycardia events</td></tr>
<tr><td>Pre-phase</td><td>0 (0, 0)</td><td>0 (0, 0)</td></tr>
<tr><td>Intervent.</td><td>0 (0, 0)</td><td>0 (0, 0)</td></tr>
<tr><td>Post-phase</td><td>0 (0, 0)</td><td>0 (0, 0)</td></tr>
<tr><td colspan="3">Pulse transit time (ms)</td></tr>
</table>
</td></tr>
</table>

Author, Year	N	Study Design	Intervention Dose (frequency, length, timing)	Mean EGA of sample Estimated PMA at intervention start	Outcomes	Results		
						Pre-phase	173.8 (165.2, 178.7)	171.7 (164.8, 182.6)
						Intervent.	175.6 (166.3, 181.5)	172.0 (164.3, 184.1)
						Post-phase	170.3 (161.6, 180.9)	173.1 (162.8, 181.8)
						**Perfusion index (%)**		
						Pre-phase	1.28 (1.19, 1.68)	1.47 (1.03, 1.70)
						Intervent.	1.46 (1.18, 1.90)	1.33 (1.09, 1.79)
						Post-phase	1.54 (1.25, 1.97)	1.33 (1.10, 1.85)
						**RMSSD (ms)**		
						Pre-phase	37.0 (28.3, 44.3)	37.1 (29.5, 51.0)
						Intervent.	37.8 (31.1, 49.5)	38.7 (31.6, 43.9)
						Post-phase	38.4 (31.6, 59.1)	36.2 (31.5, 44.8)
						**SDNN (ms)**		
						Pre-phase	31.7 (29.4, 38.6)	32.8 (25.6, 43.2)
						Intervent.	37.7 (26.5, 41.6)	35.4 (30.2, 46.6)
						Post-phase	36.4 (31.6, 48.7)	37.6 (30.3, 42.8)
						**pNN50 (%)**		
						Pre-phase	1.2 (0.7, 4.2)	1.6 (0.7, 4.2)
						Intervent.	2.2 (1.0, 4.3)	2.0 (1.0, 3.3)
						Post-phase	2.6 (1.2, 5.2)	1.7 (1.3, 2.4)

<table>
<tr><th>Author, Year</th><th>N</th><th>Study Design</th><th>Intervention Dose
(frequency, length, timing)</th><th>Mean EGA of sample
Estimated PMA at intervention start</th><th>Outcomes</th><th colspan="6">Results</th></tr>
<tr><td rowspan="6"></td><td rowspan="6"></td><td rowspan="6"></td><td rowspan="6"></td><td rowspan="6"></td><td rowspan="6"></td><td colspan="6">STAI-X1 (music, n=9; control, n=2)</td></tr>
<tr><td colspan="2">Pre-phase</td><td colspan="2">29.0 (24.5, 43.0)</td><td colspan="2">34.5 (32.0, ---)</td></tr>
<tr><td colspan="2">Post-phase</td><td colspan="2">25.0 (21.0, 36.5)</td><td colspan="2">33.0 (33.0, 33.0)</td></tr>
<tr><td colspan="6">State of the child (music, n=9; control, n=2)</td></tr>
<tr><td colspan="2">Pre-phase</td><td colspan="2">8.0 (7.0, 10.5)</td><td colspan="2">10.0 (10.0, 10.0)</td></tr>
<tr><td colspan="2">Post-phase</td><td colspan="2">7.0 (7.0, 10.5)</td><td colspan="2">11.5 (10.0, ---)</td></tr>
<tr><td rowspan="7">*Shafiei, 2020, Iran</td><td rowspan="7">40</td><td rowspan="7">Randomized crossover</td><td rowspan="7">Single group intervention of lullaby on 2 days vs. no lullaby on 2 days. Maternal lullaby recorded as the mother was alone in a room. The lullaby was played to infants via speakers for 20 min with 10 min observation before and after. On non-lullaby days, infants were observed for 40 min.</td><td rowspan="7">Included GA 28-34 weeks;

EGA 32.43±2.7 weeks; age at intervention was the third day of hospitalization</td><td rowspan="7">HR, RR, SpO_2</td><td></td><td></td><td>Before</td><td>During</td><td>After</td><td>p</td></tr>
<tr><td rowspan="2">SpO_2</td><td>Lullaby</td><td>89±3</td><td>94±5</td><td>91±2</td><td rowspan="2">.039</td></tr>
<tr><td>Control</td><td>90±1</td><td>90±2</td><td>90±2</td></tr>
<tr><td rowspan="2">RR</td><td>Lullaby</td><td>57±2</td><td>55±1</td><td>57±4</td><td rowspan="2">.070</td></tr>
<tr><td>Control</td><td>57±2</td><td>57±2</td><td>57±2</td></tr>
<tr><td rowspan="2">HR</td><td>Lullaby</td><td>154±6</td><td>149±2</td><td>151±1</td><td rowspan="2">.030</td></tr>
<tr><td>Control</td><td>156±5</td><td>156±8</td><td>155±6</td></tr>
<tr><td colspan="12">Abbreviations: BSID-III, Bayley Scales of Infant and Toddler Development, Third Edition; EGA, estimated gestational age; fMRI, functional magnetic resonance imaging; HR, heart rate, IQR, interquartile range; Lab-TAB, Laboratory Temperament Assessment Battery; NICU, neonatal intensive care unit; PMA, postmenstrual age; pNN_{50}, proportion of the number of interval differences of successive normal-to-normal intervals >50 ms divided by the total number of normal-to-normal intervals; RMSSD, root mean square of successive differences; RR, respiratory rate, SDNN, standard deviation of normal-to-normal; SpO_2 oxygen saturation; STAI-X1, State Trait Anxiety questionnaire.</td></tr>
</table>

3. Table of vision sensory studies

Author, Year	N	Study Design	Intervention Dose (frequency, length, timing)	Mean EGA of sample and Estimated PMA at intervention start	Outcomes	Results
Morag 2017	Cochrane review	*all articles in this Cochrane review on cycled light were included in the previous integrative review				All the studies in this updated Cochrane review were part of our previous review;
Kaneshi 2016	21 white light; 21 red light	Randomized controlled trial	All infants were exposed to cycled light (15h light/9h dark). During nighttime nursing care (feeding and diaper changes), infants in were exposed to either a red or white LED light.	Mean EGA 32.3±2.2 in white light group and 32.4±1.8 in red light group; PMA at the start of the intervention was 36.1±1.3 in white light group and 35.8±1.8 in red light group	Infant activity (absolute number of movements per hour), nighttime crying episodes, and weekly weight gain	**Activity:** no difference between groups during daytime or nighttime activity; at 35 weeks, day-night activity ratios were 1.06±0.13 for white light and 1.24±0.47 red light, indicating more activity during the day for red light infants, but day-night activity ratios over time were not significantly different between groups (p>.05) **Nighttime crying:** not different between white and red light groups (p>.05) **Weight gain:** no differences between white and red light groups (p>.05)
**Brandon 2017, USA	61 early cycled light, 57 late cycled light	Randomized controlled trial	All infants first received near darkness, and then cycled light implemented early (28 weeks PMA) or late (36 weeks PMA) via bed covers and dimmed light; near darkness (5-30 lux) provided for 11-12h (depending on shift change needs) and daylight (200-600 lux) for 11-12h	Enrolled infants< 28 weeks EGA; Mean EGA 26.3±1.4 in early cycled light and 26.3±1.5 in late cycled light groups; intervention started at 28 weeks and 36 weeks PMA for early and late cycled light groups, respectively	Weight gain (in-hospital and outpatient), LOS, hospital costs, sleep development (sleep-wake coding system) in-hospital and outpatient (measured at 4 months until 24 months by parent-report sleep diaries), neurodevelopmental outcomes (Bayley-II, Preferential Looking Test)	**In-hospital weight gain (mean, g):** no difference (early 193.8 vs. late cycled light 176.3) **Outpatient weight gain (mean, g):** no difference (p=.418) **LOS (mean, days):** no difference (early 91.4±45.0 vs. late cycled light 96.9±48.2, p=.586) **Hospital cost (mean, USD):** no difference (early $127,650±75,744

Author, Year	N	Study Design	Intervention Dose<br><br>(frequency, length, timing)	Mean EGA of sample and<br><br>Estimated PMA at intervention start	Outcomes	Results
						vs. late cycled light $140,501± 93,260, p=.446)<br><br>**In-hospital sleep development:** most sleep-wake states were not different between intervention groups; active sleep at night showed a significant interaction between time and intervention (p=.04) "suggesting the rate of decrease in active sleep was accelerated at 35-36 weeks of PMA for infants receiving [early cycled light] compared to those in the [late cycled light] group." Waking during the day was increased in infants in the late cycled light group (p=.018).<br><br>**Outpatient sleep development:** no difference in sleep bouts during the day (p=.615) or night (p=.087); longest sleep bouts in 24 hours were longer in the late cycled light vs. early cycled light group (p=.041); variability of bedtime was not different between groups (p=.140).<br><br>**Neurodevelopmental outcomes:** no difference between intervention groups in any neurodevelopmental outcome at 9, 18, or 24 months.
Tandircioglu 2019, Turkey	11 blanket on incubator all the time,	Randomized controlled trial	In one group, incubators were covered with a blanket all day. In the second group, incubators	Mean EGA 32.5±1 in near darkness, 32±1 in no darkness, and 32±1.2 in cycled light groups;	Sleep-wake cycle pattern (measured by aEEG) for 24h on third and tenth day of the	**Sleep-wake cycle (median, mV):** narrowband lower amplitudes in the blanket all

Author, Year	N	Study Design	Intervention Dose (frequency, length, timing)	Mean EGA of sample and Estimated PMA at intervention start	Outcomes	Results
	11 no blanket on incubator, 10 blanket on and off incubator in 12 hr cycles		were never covered. In the third group, incubators were covered for 12 hours and uncovered for 12 hours.	no information on timing of intervention	study, Burdjalov scores (range from 0 to 20)	day group (10mv) on the third day of the study (7mV for no blanket and 12h blanket, p=.042), but differences were not observed on the tenth study day (blanket all day 10mV vs. no blanket 7.5mV vs. 12h blanket 8mV, p=.110)<br><br>**Burdjalov scores (median):** data were not reported according to intervention group. Infants 30-31 weeks had lower scores (9.0) vs. 32-33 weeks (10.0) and 34-35 weeks (11.0, p=.014) on the third study day. Scores remained higher for infants at 34-35 weeks (12.0) on the tenth study day compared to 30-31 week (10.0) and 32-33 week infants (10.0, p=.007).
Lebel 2017, Canada	16 cycled light; 20 continuous near darkness	Randomized controlled trial	Cycled light group exposed to 200-225 lux between 7am and 7pm and <20 lux between 7pm and 7am determined by raising and lowering an incubator cover; continuous near darkness group exposed to <20 lux for the full day; exposure to the assigned intervention lasted for 24h only	Mean EGA 30.04±1.28 in near darkness group and 30.21±1.29 in cycled light group; intervention start when infants were >24h. Approximate timing of intervention was 28 weeks PMA to term	Physiologic stability (SCRIP score, RR, HR, SpO_2), motor activity (accelerometer measured the presence or absence of motor activity in 15 min intervals)	**SCRIP for 24h (mean):** no difference (cycled light 5.84±0.27 vs. near darkness 5.84±0.19, p=.96)<br><br>**RR for 24h (mean):** no difference (cycled light 53.91±11.69 vs. near darkness 51.02±6.61, p=.39)<br><br>**HR for 24h (mean):** no difference (cycled light 161.02±9.49 vs. near darkness 162.47±7.10, p=.60)<br><br>**SpO2 for 24h (mean):** no difference (cycled light 95.16±2.94 vs. near darkness 94.88±3.13, p=.81)

Author, Year	N	Study Design	Intervention Dose<br>(frequency, length, timing)	Mean EGA of sample and<br>Estimated PMA at intervention start	Outcomes	Results
						**Activity periods for 24h (mean):** no difference (cycled light 1640.8±416.14 vs. near darkness 1656.05±485.11, p=.84)<br><br>**Duration of handling (mean, minutes):** no difference (cycled light 94±39.68 vs. near darkness 116.85±44.45, p=.12)<br><br>**Frequency of positioning (mean):** no difference in dorsal (cycled light 2.25±2.02 vs near darkness 2.15±1.14, p=.31), ventral (cycled light 2.13±1.63 vs. near darkness 2.15±1.35, p=.79), right side (cycled light 1.75±0.68 vs. near darkness 2.30±1.22, p=.26), or left side (cycled light 2.25±1.13 vs. near darkness 1.70±1.13, p=.16)<br><br>**Kangaroo care:** no difference in duration (cycled light 93.81±104.31 min vs. near darkness 59.2±68.26 min, p=.24) or frequency (cycled light 1±1.10 vs. near darkness 0.7±0.8, p=.71)<br><br>**Noise intensity (mean, dBA):** no difference (cycled light 51.16±3.92 vs. near darkness 52.48±4.0, p=.33)<br><br>No differences when stratified by day and night.

Abbreviations: aEEG, amplitude-integrated electroencephalograph; HR, heart rate; LOS, length of stay; PMA, postmenstrual age; RR, respiratory rate; SCRIP, Stability of the Cardiorespiratory System in Premature Infants; SpO_2, oxygen saturation.

4. Table of kinesthetic sensory studies

Author, Year	N	Study Design	Intervention Dose (frequency, length, timing)	Mean EGA of sample and Estimated PMA at intervention start	Outcomes	Results
*Litmanovitz, 2016, Israel	13 twice daily exercise, 12 once daily exercise, and 11 no intervention	Randomized controlled trial	Intervention was passive extension and flexion range of motion exercise of the upper and lower extremities performed for 10 min per session 5 times per week for 4 weeks. Intervention was performed either once or twice daily. Controls received 10 min per day interactive periods of holding and stroking without range of motion activity.	Mean EGA was 28.5±1.4 for twice daily intervention, 28.8±0.8 for once daily intervention, and 28.9±1.2 for controls; intervention started at 8±2.4 days of life. Intervention was done from approx. 28 weeks PMA to 36 weeks PMA.	Left tibial speed of sound (measure of bone strength) at study entry and 2 and 4 weeks later; growth parameters (weight, length, head circumference)	**Weight (mean, g):** no difference at 2 weeks (twice daily 1410±208 vs. once daily 1470±254 vs. control 1451±234, p=.75) or 4 weeks (twice daily 1834±289 vs. once daily 1883±340 vs. control 1941±337, p=.72)<br><br>**Length (mean, cm):** no difference at 2 weeks (twice daily 40.3±1.9 vs. once daily 40.5±1.4 vs. 40.0±1.7, p=0.79) or 4 weeks (twice daily 42.0±1.7 vs. once daily 42.2±1.7 vs. control 42.4±1.7, p=.9)<br><br>**Head circumference (mean, cm):** no difference at 2 weeks (twice daily 28.2±1.3 vs. once daily 28.4±1.6 vs. control 28.7±1.3, p=.66) or 4 weeks (twice daily 30.2±1.3 vs. once daily 29.8±1.3 vs. control 30.4±1.4, p=.77)<br><br>**Tibial speed of sound (mean, m/s):** no difference at baseline (twice daily 2918±78 vs. once daily 2943±119 vs. control 2910±48); rate of decline was lower for twice daily exercise (2894±105) vs. control (2800±85, p=.026) at 4 weeks but not significantly different from controls for once daily exercise group (2875±94)
*Sezer Efe, 2020, Turkey	12 exercise, 12 control	Randomized controlled trial	Daily range of motion exercises and extension and flexion in upper and lower extremities administered 5-8 times, one session per day	Mean EGA was 30.58±1.56 in exercise group and 30.75±1.42 in control group; intervention started at 31.83±1.69 PMA in exercise group and 33.25±1.95 PMA in control group	Tibial speed of sound, growth parameters (weight, length, head circumference, chest circumference, waist circumference, right tibial length, mid-upper arm	**Tibia speed of sound (mean, m/s):** no difference at baseline after power analysis revealed appropriate sample size (exercise 2959.75±117.89 vs. control 3000.33±106.52, p=.386); higher for exercise group after intervention (exercise 2985.41±96.08 vs. control 2870.58±99.29, p=.009)

Author, Year	N	Study Design	Intervention Dose (frequency, length, timing)	Mean EGA of sample and Estimated PMA at intervention start	Outcomes	Results
			for 30 days. Each session lasted 7-10 min.		circumference, triceps skinfold thickness)	**Weight (mean, % increase):** exercise 40.58±17.57 vs. control 39.13±16.94, p=.838<br><br>**Length (mean, % increase):** exercise 13.57±6.91 vs. control 12.42±5.11, p=.648<br><br>**Head circumference (mean, % increase):** exercise 12.93±3.68 vs. control 12.74±5.38, p=.917<br><br>**Chest circumference (mean, % increase):** exercise 15.32±5.69 vs. control 13.69±8.18, p=.577<br><br>**Waist circumference (mean, % increase):** exercise 10.83±4.09 vs. control 10.54±5.83, p=.887<br><br>**Right tibial length (mean, % increase):** exercise 24.32±7.94 vs. control 18.11±7.89, p=.068<br><br>**Mid-upper arm circumference (mean, % increase):** exercise 20.09±6.61 vs. control 25.57±9.49, p=.115<br><br>**Triceps skinfold thickness (mean, % increase):** exercise 29.85±14.60 vs. control 32.21±17.86, p=.726
** Ustad 2016, Fjørtoft 2017, Øberg 2020, Norway	153 total; 71 physical therapy, 79 controls (fewer at follow-up)	Randomized controlled trial	Parent-administered physical therapy (guided movement to improve postural control) with the infant in various positions. The intervention was individualized for the infant's development level and tolerance. Conducted for 10 min twice per day for 3 weeks.	All infants EGA $\leq$32 weeks (14% physical therapy and 19% control group were <28 weeks); intervention was administered at PMA 34, 35, and 36 weeks	TIMP/TIMPSI at baseline, 37 weeks PMA, and 3 months corrected age; fidgety movements (present or absent), temporal organization of fidgety movements, and movement character at 3 months corrected age	**TIMP/TIMPSI**<br><br>*Ustad 2016 (mean z-score[a]):* No difference between groups at baseline (physical therapy 0.07 [95% CI -0.19, 0.34] vs. control -0.07 [95% CI -0.27, 0.13], p=.394); higher for physical therapy group at 37 weeks (physical therapy 0.21 [95% CI -0.02, 0.45] vs. control -0.18 [95% CI -0.42, 0.06], p=.005)<br><br>*Øberg 2020 (mean clinical z-score[a])*: No difference at baseline (physical therapy −0.32 [95% CI −0.45, −0.18] vs. control −0.42 [95% CI −0.54, −0.30], p=.43). At 37 weeks physical therapy had higher clinical z-scores (0.03 [95% CI −0.12, 0.19] vs.

Author, Year	N	Study Design	Intervention Dose (frequency, length, timing)	Mean EGA of sample and Estimated PMA at intervention start	Outcomes	Results
						controls (−0.24 [95% CI −0.39, −0.08], p=.014). At 3 months, there was no difference between groups (physical therapy −0.04 [95% CI −0.20, 0.12] vs. controls − 0.08 [95% CI −0.23, 0.06], p=.57). Adjusted analysis showed a positive relationship with total dose of physical therapy at 3 months; longer intervention times increased TIMP scores (beta-coefficient 0.14, p=.003).<br><br>**Fidgety movements (present vs. absent):** no difference in presence of fidgety movements (physical therapy 48/59 [81%] vs. control 60/71 [85%], p=.647)<br><br>**Temporal organization of fidgety movements:** no difference in continual (physical therapy 11/59 [19%] vs. control 15/71 [21%]), intermittent (physical therapy 37/59 [63%] vs. control 45/71 [63%]), sporadic (physical therapy 8/59 [14%] vs. control 9/71 [13%], or absent (physical therapy 3/59 [5%] vs. control 2/71 [3%], p=.912)<br><br>**Movement character (smooth and fluent vs. abnormal, not cramped-synchronized):** no difference in smooth and fluent movements (physical therapy 25/59 [42%] vs. control 38/71 [54%], p=.222)

Abbreviations: CI, confidence interval; EGA, estimated gestational age; OR, odds ratio; PMA, postmenstrual age; TIMP, Test of Infant Motor Performance; TIMPSI, Test of Infant Motor Performance Screening Items.

a. Different z-score calculations were used by Ustad 2016 and Øberg 2020.

5. Table of olfactory and gustatory sensory studies

Author, Year	N	Study Design	Intervention Dose (frequency, length, timing)	Mean EGA of sample and Estimated PMA at intervention start	Outcomes	Results
Russell, 2015, USA	11 control, 11 unscented simulation device, 11 maternal scented simulation device, 12 scented nesting device	Randomized trial	Standard care (control): infant extremities in flexed position, close to body; Unscented simulation device: unscented device that mimics holding; Scented simulation device: maternally-scented device that mimics holding; Scented nesting device: padded bumper/quilted fleece pad for positioning and containment. Dose not reported but infants observed every 2 hours for 12 consecutive hours.	Mean EGA was 30.5±4.6 for controls, 30.1±3.7 for unscented simulation device, 31.0±3.9 for scented simulation device, and 28.1±4.0 for scented nesting device; mean day of life at intervention was 18.9±23.9 for controls, 15.1±19.7 for unscented simulation device, 6.7±6.4 for scented simulation device, and 17.3 for scented nesting device. Intervention occurred from 24-38 weeks PMA.	Cardiorespiratory events (apnea or bradycardia), premature infant pain profile (7-item, 4-point scale), self-regulatory indicators (cardiorespiratory stability, color, feeding tolerance, smooth body movements, well-defined sleep/wake states, self-quieting behaviors, attentive behaviors)	**Premature infant pain profile:** data were not reported in the study but stated there were no significant differences between the study groups<br>**Cardiorespiratory events (intervention vs. controls):**<br>Unscented simulation: OR 0.095, 95% CI 0.027, 0.337<br>Scented simulation: OR 0.031, 95% CI 0.004, 0.236<br>Scented nesting: OR 0.482, 95% CI 0.222, 1.293<br>**Color (intervention vs. controls):**<br>Unscented simulation: OR 0.803, 95% CI 0.380, 1.701<br>Scented simulation: OR 0.295, 95% CI 0.120, 0.728<br>Scented nesting: OR 0.662, 95% CI 0.312, 1.404<br>**Feeding (intervention vs. controls):**<br>Unscented simulation: not calculable<br>Scented simulation: not calculable<br>Scented nesting: OR 2.026, 95% CI 0.558, 7.32<br>**Body movements (intervention vs. controls)**<br>Unscented simulation: OR 0.099, 95% CI 0.044, 0.222<br>Scented simulation: OR 0.060, 95% CI 0.025, 0.141<br>Scented nesting: OR 0.219, 95% CI 0.102, 0.470

Author, Year	N	Study Design	Intervention Dose (frequency, length, timing)	Mean EGA of sample and Estimated PMA at intervention start	Outcomes	Results
						**Sleep/wake state (intervention vs. controls):**<br>Unscented simulation: OR 0.096, 95% CI 0.042, 0.221<br>Scented simulation: OR 0.110, 95% CI 0.048, 0.252<br>Scented nesting: OR 0.449, 95% CI 0.197, 1.025<br>**Self-quieting (intervention vs. controls):**<br>Unscented simulation: OR 0.651, 95% CI 0.327, 1.297<br>Scented simulation: OR 0.301, 95% CI 0.143, 0.632<br>Scented nesting: OR 1.177, 95% CI 0.602, 2.301<br>**Attentive behaviors (intervention vs. controls):**<br>Unscented simulation: OR 0.565, 95% CI 0.280, 1.142<br>Scented simulation: OR 0.155, 95% CI 0.072, 0.334<br>Scented nesting: OR 0.474, 95% CI 0.238, 0.947

Abbreviations: CI, confidence interval; EGA, estimated gestational age; OR, odds ratio; PMA, postmenstrual age.

6. Table of multimodal sensory studies.
   *Note that studies in this section evaluated an intervention that included more than one type of sensory exposure. In addition, studies that evaluated one type of sensory exposure to another (across categories) is included here, however, it may be reported differently in the results and PMA tables based on the findings.

Author, Year	N	Study Design	Intervention	Estimated GA	Outcomes	Results

			Dose<br>(frequency, length, timing)	Mean EGA of sample<br>Estimated PMA at intervention start		
**Baby 2015<br>India	60	Randomized Controlled Trial	Tactile + Kinesthetic stimulation vs control<br>15 min sessions, 3x per day, 30 min after feeding, for 10 days	28-34 weeks GA; no average GA was reported; 43% were between 32-34 GA; 30% were between 30-32 GA and 27% were between 28-30 GA<br>Unclear what PMA	Level of neuromuscular maturity (Ballard's Maturational Assessment Scale)	There was a significant difference in neuromuscular maturity in intervention group from pre-test to post-test (17.7 vs 26.57) compared to control group (17.53 vs 22.53, p=0.002).
*Aldana-Acosta 2019<br>Columbia	66	Randomized Controlled Trial	Kinesthetic stimulation while in kangaroo position vs kinesthetic stimulation in incubator<br>Kinesthetic stimulation (Field protocol) with sunflower oil, 3x per day, for 15 consecutive days in hospital or home	30-33 weeks GA<br>32.1±1.0 weeks GA kangaroo position group; 32.2±0.8 weeks GA incubator group<br>Chronological age at intervention start 7.7±4.3 kangaroo position; 7.2±4.4 incubator (assumed days)	Weight gain at 5 days, and 15 days and 40 weeks	Daily weight gain was significantly higher with kangaroo position compared to incubator at 5 days (11.0 g/kg/day 95% CI 5.7-16.3 vs 2.1 g/kg/day 95% CI 3.1-7.4, p=0.02) and at 15 days (12.1g/kg/day 95%CI 10.4-13.7 vs 9.4g/kg/day 95%CI 7.7-11.1)<br>Weight at 40 weeks was higher in kangaroo position group (2904 g) than incubator group (2722g, p=0.05)
Baniasadi 2019<br>Iran	45	Quasi-experimental	One group measured before and after massage intervention	26-34 weeks GA	Behavioral state: sleep state, awake state, motor activity and behavioral distress	There were significant differences in behavioral components of sleep state (p=0.003), awake state (p=0.04), fidgeting/crying (p=0.03), and motor activity (p=0.001), from pre- to post-massage.

			(tactile + kinesthetic stimulation)<br><br>15 min sessions, once a day, 5 days	Mean GA 32.96 weeks		No significant difference in total behavioral distress between pre and post massage
**Vahdati 2017<br><br>Iran	64	Randomized controlled trial	2 groups: kangaroo care + music vs kangaroo care<br><br>Kangaroo care for 60 min per day, 3 consecutive days; music provide for 20 min during KC	Mean GA: 32.74±1.81 weeks (KC + music)<br><br>33.04±1.98 weeks (KC)	Mother-infant attraction (Avant's Maternal Attachment Scale)	Mean overall attraction (70.72±11.46 vs 53.61±9.76, p<0.001), emotional behaviors (77.19±12.64 vs 62.90±12.13, p<0.001), Proximity behaviors (65.88±15.02 vs 44.53±12.27, p<0.001), and care behaviors (60.67±17.61 vs 41.66±14.19, p<0.001) were higher in the KC + music group compared to KC only group
Ettenberger 2017<br><br>Colombia	36	Non-randomized Trial	2 groups: Music therapy during kangaroo care vs control<br><br>Intervention: parents sat in chairs holding infants in Kangaroo Care, music therapist then sat in front and played with instrument and sang together with parents, 2x/week, until discharge, session duration ranged from 10-40 min	GA: 28-34 weeks<br><br>Mean GA: 32.1 (MT group)<br><br>32.0 (controls)<br><br>Interventions started at ≥30 weeks GA	Parental anxiety (State-Trait Anxiety Inventory), parent-infant bonding (Mother-to-Infant-Bonding Scale), heart rate, oxygen saturation, weight gain, length of hospitalization, re-hospitalization rate	**Anxiety:** there were significant improvements in maternal anxiety (p=0.007)<br><br>Positive trends were found in length of hospitalization and re-hospitalization rate, but were not statistically significant<br><br>**Weight gain**: a significant increase in weight gain per day was found for MT group vs controls (24.2g/day vs 18.5g/day, p=0.036)
*Alvarez 2019<br><br>Spain	136	Quasi-experimental	Massage therapy + kinesiotherapy (Field) vs control (standard care)<br><br>15 min sessions, once a day, given by parents,	<37 weeks GA<br><br>Mean GA 32.1 ±4.1 weeks	Weight, head circumference	**Weight:** infants in the massage therapy group gained more weight (mean weight gain 895.7 ±547.9 grams) than control infants (541.8±536.2 grams)<br><br>**Head circumference**: infants in massage therapy group grew more in head circumference (mean increase in

			unclear length of intervention			circumference 4.2±3.2 cm) than control infants (2.4±2.6 cm)<br><br>**Length**: massage therapy grew more in length (mean increase in length 5.5±4.3 cm) than control infants (3.0±3.1 cm)
Carvalho 2019<br><br>Portugal	36 mother-infant dyads	Single group repeated measures	1 group: 15 min session of skin-to-skin contact. The first 3 min (baseline) were in silence, the next 3 min mothers were invited to sing or speak to baby, then 3 min of quiet (offset period), followed by another 3 min of singing or speaking (opposite of previous singing or speaking period), and a final 3 min of silence<br><br>Group 1: sing first, then speak<br><br>Group 2: speak first, then sing	Mean GA: 30 weeks<br><br>intervention at 32-37 weeks<br><br>Mean chronological age: 26.5 days	Number of infant vocalizations from coded videos; frequency of vocalizations; patterns of vocalizations in relation to parent	A total of 1172 infant vocalizations were identified, 306 of which were vocalizations during the baseline periods.<br><br>A total of 2015 vocalizations were recorded during the speaking interventions, and 189 during the singing intervention<br><br>There was a significantly higher frequency of maternal vocalizations during speaking intervention compared to singing intervention<br><br>Compared to baseline (mean=8.5), infants vocalized less frequently during the singing intervention (mean=5.61, p=0.042) than during the speaking intervention (mean=5.66, p=0.049<br><br>Compared to baseline, infants vocalized less frequently during singing offset period (p=0.036) but not significantly different in speaking offset.<br><br>No significant difference was found in infant vocalizations frequency between singing and speaking conditions<br><br>Overlapping vocalizations in the singing (mean=4.30) period was higher than overlapping vocalizations in the speaking period (mean=2.39, p=0.007)<br><br>Infant vocalizations occurred more frequently during pauses in speaking (mean=3.03) than during pauses in singing (mean=1.30, p=0.004)
*Choi 2016<br><br>South Korea	20; had a power analysis	Non-randomized Trial	2 groups: massage therapy vs control	30-34 weeks GA	Height, weight, head circumference, chest circumference, ab-dominal circumfer-	**Height:** was significantly increased in the massage group from week 1 to week 2, but not in control group

			Massage was performed in 15 sessions, twice per day, for 14 days	Mean GA: 31.6 weeks (massage)<br><br>31.4 weeks (control)	ence, and gastrointestinal function	**Head circumference**: slight increase in both groups over time, but not statistically significant<br><br>**Chest circumference:** significant increase in both groups over time, but larger increase in massage group.<br><br>**Abdominal circumference**: slight increase in both groups over time, but not statistically significant<br><br>**Gastrointestinal function:** frequency of pre-feed gastric residual was significantly decreased for both groups and numbers of bowel movements were significantly increased in the massage group.
**Detmer 2020	48	Randomized Controlled Trial	2 groups: Multimodal Neurologic Enhancement vs control Intervention: consisted of auditory stimulation: gentle quiet live lullaby singing in the key of A,C, or D; tactile stimulation: stroking each area of infant's body with 1-2 fingers in firm but gentle manner; vestibular stimulation: front-to-back rocking in rocking chair in synchrony to tempo of the music; visual stimulation: eye contact from music therapist. 20 min sessions, 2-3 times per week, for the duration of NICU stay	GA: <32 weeks<br><br>Intervention started as early as PMA 32 weeks	Early Learning Composite score	**Visual:** Experimental group performed significantly better than control group on the Visual Reception (mean 50.7 vs 45.36, respectively p=0.016)<br><br>**Early Learning Composite scores:** experimental group also performed significantly better than control in composite score (mean 102.7 vs 95.40, respectively p=0.031)<br><br>No significant differences in gross motor, fine motor, receptive language, or expressive language scores
Efendi 2018<br><br>Indonesia	84	Randomized Controlled Trial	4 groups: Therapeutic touch vs voice stimulus vs therapeutic touch +	GA: < 37 weeks<br><br>Mean GA: 32.68 weeks (MTT)	Heart rate, respiratory rate, oxygen saturation, quality of sleep,	**Heart rate:** the MVS group showed a significant change in mean HR, 5.63 higher than in the control group (p=0.0001). MTT and MTT + MVS groups did not show

			voice stimulus vs control (routine care)<br><br>Therapeutic Touch: slanted infant's body to one side, flexed body, positioned hands close to mouth and flexed legs closer to the tummy, then placed hand above infant's neck and head while resting the other on lower abdomen. Performed by nurse and mother. 15 min session<br><br>Voice stimulus: recording of each mother singing the "Nina Bobo" lullaby song for 15 min, volume set to < 65 dB, played 10-15 cm from infant's head	32.02 weeks (MVS)<br><br>MTT + MVS unclear<br><br>31.80 weeks (control)<br><br>Chronological age: 2-30 days		significant increase in HR (p=0.353 and p=0.085, respectively)<br><br>**Respiratory Rate:** MTT group showed changes in respiratory rate 2.3 times higher than control group (p=0.001) and MVS group showed significant changes in respiratory rate 1.84 times higher than control (p=0.008). MTT + MVS group showed a decrease in HR (B=0.46) but was not significantly different from control group (p=0.509)<br><br>**Oxygen saturation:** average SpO2 in the MTT group was 2.38 times lower than in the control group (p=0.001). control group experiences a decrease in oxygen saturation during prick phase, but other groups showed stable oxygen saturation during suctioning. MTT + MVS group showed the most stable SpO2 from baseline, therapy, and recovery phases.<br><br>**Infant sleep scores:** infants in MVS group had a significant increase in sleep score (1.7 time higher than control group, p=0.0001). infants in MTT + MVS group also had significant increase in sleep score (0.4 times higher than controls, p=0.01). no significant increase was seen in sleep scores for MTT group (p=0.202). meaning the most stable sleep states were seen in MTT infants. Sleep scores increased significantly in all groups during prick intervention. MTT showed the lowest scores for sleep state at this phase and was the fastest group to go back to sleep.
*El-Farrash 2019<br><br>Egypt	36	Randomized controlled trial	2 groups: range of motion exercises with gentle compression, extension, and flexion of all joints of both the bilateral upper and lower extremities, 10 min session per day, 5 days a week, 4 weeks vs tactile stimulation consisting of holding and stroking but no	GA: ≤32<br><br>Chronological age: 7 days	Serum calcium, serum phosphorus, magnesium, alkaline phosphatase, urinary calcium/phosphate ratio, and serum carboxyterminal cross-linked telopeptide of type 1 collagen, bone mineral content, bone mineral density, bone	**Serum calcium:** no significant difference in groups after intervention<br><br>**Serum phosphorus:** exercise group had higher levels (p=0.001)<br><br>**Magnesium:** no significant difference between groups after intervention<br><br>**Alkaline phosphate:** exercise group had significantly lower ALP levels (p=0.005)

			range-of-motion activity, once daily		area, lean mass, and fat mass	**Urinary calcium/phosphate ratio:** exercise group had significantly lower levels following intervention (p=0.04)<br><br>**Serum carboxyterminal:** no significant difference between groups after intervention<br><br>**Bone mineral content:** no significant difference<br><br>**Bone mineral density:** exercise group had slightly higher bone mineral density following intervention (0.5 g/cm2) compared to controls (0.4 g/cm2, p<0.001)<br><br>No significant differences in bone area, lean mass, or fat mass<br><br>**Weight gain:** rate of weight gain was significantly higher in exercise group (34.7g/day) compared to controls (17.6 g/day, p<0.001)
**Konar 2019<br><br>India	2294	Randomized Controlled Trial	2 groups: body massage with coconut oil vs body massage with no oil<br><br>Intervention: application of 5ml of coconut oil all over the body, excluding face and scalp, by gentle massaging, 4x daily	Mean GA: 31.9 ±3.4 weeks; says intervention done in the hospital and after discharge but unclear what starting PMA is	Neonatal skin condition (Neonatal Skin Condition Score), neurodevelopmental status (DASII scale), weight gain, weight loss, serum vitamin D levels	**Weight gain:** Mean weight gain per day was higher in massage only group (1.21% vs 0.89%, p<0.01)<br><br>**Weight loss:** Mean weight loss was significantly less in oil massage group (4.71% vs 7.82%, p<0.001**).** Incidence of hypothermia (1.9% vs 5.8%, p<0.01) and apnea (1.2% vs 5.1%, p<0.01) was significantly lower in oil massage group compared to massage only group**.**<br><br>**Vitamin D:** mean serum D3 level on day 30 was higher among oil massage group than massage only group (32.3 vs 24.6 ng/ml, p<0.01)<br><br>**Skin condition:** NSCS was significantly better in oil massage group than massage only group on day 7, 14, 21 and 28 (p<0.01)<br><br>**Neurodevelopment:** both motor and mental developmental quotients were significantly higher in oil massage group than massage only group at 3, 6, and 12 months (p<0.01).<br><br>Infants in oil massage group were 0.31 (95% CI 0.24-0.39) and 0.59 (95% CI 0.45-0.74) times less likely to

						suffer from decreased skin maturity and adverse neurodevelopmental outcome, respectively.
*Taheri 2018<br><br>Iran	44	Randomized Controlled Trial	2 groups: massage with sunflower oil vs control (standard NICU care)<br><br>Intervention: 3x per day, each session with 3 consecutive 5-min stages (15min total), 5 days. First 5 min infant was placed in prone position and massaged with moderate pressure and 10 cc/kg/day of sunflower oil, in the second 5 min in infant was placed in supine position and passive extension/flexion movements were performed, in the last 5 min the infant was placed back in prone position and massage from the first phase was repeated	Corrected GA: 30-36 weeks<br><br>Mean corrected GA: 32.63±1.75 weeks<br><br>Mean GA: 30.64±2.13 weeks (oil massage)<br><br>30.53±1.72 weeks (control)	Weight gain, length of NICU stay,	**Weight gain:** average daily weight gain was significantly higher in the oil massage group compared to controls (14.90±8.29 g/day vs -14.90±37.88, p=0.001)<br><br>**NICU stay:** mean length of NICU stay was significantly shorter for oil massage group compared to controls (24.5±13.2 days vs 36.27±10.2 days, p=0.032)
*Elmoneim, 2021, Egypt	60	Randomized controlled trial	Massage therapy: 3 consecutive 15 min sessions per day after the noon feeding which included 5 min tactile stimulation, 5 min kinesthetic stimulation, and 5 min tactile stimulation. Intervals of	Included GA <32 weeks; EGA 30.7 ± 1.3 weeks for massage group and 31.0 ± 1.3 weeks for controls; intervention started at 35 weeks PMA	Both cumulative and daily measures of weight gain, growth velocity, length, ponderal index, head circumference, mid-arm circumference, thigh circumference, body composition pa-	Note: All anthropometric measures are median, IQR. Only cumulative measures are shown here as findings were similar for daily measures.<br><br>**Cumulative weight gain (g):** higher for massage (77.5, 40-137) vs. routine care (25, 10-73, p=.003)<br><br>**Cumulative length gain (cm):** higher for massage (0.50, 0-1) vs. routine care (0, 0-0.5, p=.02)

<table>
<tr>
<td></td>
<td></td>
<td></td>
<td>45 min occurred between sessions.

Routine care: observed at a single time point (unclear if recorded daily or on multiple days)</td>
<td></td>
<td>rameters from DXA scan (total body less head, arms, legs, trunk), temperature, episodes of hypothermia, blood pressure, episodes of hypo/hypertension, HR, episodes of tachy/bradycardia, SpO_2, episodes of desaturation and apnea</td>
<td>Cumulative ponderal index gain (g/cm3): not different for massage (2.6, 1.6-4.6) vs. routine care (2.3, 1.2-3.4, p=.77)

Cumulative head circumference gain (cm): not different for massage (0.5, 0-0.8) vs. routine care (0.5, 0.5-1.5, p=.84)

Cumulative mid-arm circumference gain (cm): higher for massage (0.5, 0.5-0.5) vs. routine care (0, 0-0.5, p=.04)

Cumulative thigh circumference gain (cm): not different for massage (0.5, 0.2-0.6) vs. routine care (0.5, 0-0.6, p=.28)

Note: DXA measures are mean ± SD. Only percent change comparisons are reported here.
<table>
<tr><td></td><td>Massage</td><td>Rout. Care</td><td>p</td></tr>
<tr><td colspan="4">Total Body Less Head DXA</td></tr>
<tr><td>Tissue mass (kg)</td><td>19.8 ± 6.1</td><td>4.2 ± 1.2</td><td><.001</td></tr>
<tr><td>Fat mass (g)</td><td>64.1 ± 23</td><td>27 ± 12</td><td><.001</td></tr>
<tr><td>Lean mass (g)</td><td>15.2 ± 5.6</td><td>10.6 ± 4.1</td><td>.001</td></tr>
<tr><td>BMD (g/cm^2)</td><td>5.8 ± 1.3</td><td>5.9 ± 1.4</td><td>.775</td></tr>
<tr><td colspan="4">Arms DXA</td></tr>
<tr><td>Fat (g)</td><td>11.2 ± 4.7</td><td>10.2 ± 1.9</td><td>.284</td></tr>
<tr><td>Lean mass (g)</td><td>61.8 ± 13</td><td>27.8 ± 11</td><td><.001</td></tr>
<tr><td>BMD (g/cm^2)</td><td>37.2 ± 11</td><td>6.1 ± 2.3</td><td><.001</td></tr>
<tr><td colspan="4">Legs DXA</td></tr>
<tr><td>Fat (g)</td><td>61.1 ± 13</td><td>21.2 ± 11</td><td><.001</td></tr>
</table>
</td>
</tr>
</table>

						Lean mass (g)	56.2 ± 17	17.6 ± 11	<.001
						BMD (g/cm²)	36.1 ± 12	6.2 ± 2.2	<.001
						**Trunk DXA**			
						Fat (g)	17.4 ± 7.1	21.1 ± 9.9	.102
						Lean mass (g)	19.1 ± 8.3	16.1 ± 7.7	.152
						BMD (g/cm²)	22.1 ± 8.8	6.7 ± 3.5	<.001
						Note: physiological measures are mean ± SD or N(%). Comparisons use data obtained during massage vs. a single time point for routine care.			
						**Temp (°C)**	36.7 ± 0.4	36.8 ± 0.4	.34
						**BP (mmHg)**	39.6 ± 1.6	39.3 ± 1.2	.41
						**HR (bpm)**	155.1 ± 24	134.4 ± 29	.004
						**Bradycardia**	2 (7%)	0	.49
						**SpO_2 (%)**	92.3 ± 2.5	93.4 ± 1.8	.06
						**Desaturation**	4 (13%)	0	.11
						**Apnea**	2 (7%)	0	.10
						No episodes of hypothermia, hypo/hypertension, or tachycardia in both groups			
Epstein, 2020,<br><br>Israel	35 (with IVH grades 3 or 4 or PVL)	Randomized crossover	Each infant received 3 sessions of maternal singing combined with SSC and 3 sessions of SSC alone in alternating order. Sessions occurred 2 to 3 times per week, beginning with 10 min of SSC alone, followed by 20 min of SSC alone or SSC with maternal	Included GA<32 weeks;<br><br>EGA 27 ± 2.5 weeks;<br><br>PMA at intervention 31/32 weeks	HR variability (low/high frequency ratio), infant and maternal vital signs (HR, RR, SpO_2), maternal anxiety (state-trait anxiety inventory), and infant behavioral state (Als criteria, range 1 to 7)	**HR variability (mean±SD, low/high frequency ratio):** Less in the SSC group (1.1±0.25) vs. SSC + maternal singing (1.8±0.7, p=.01)<br><br>**Infant HR (mean±SD):** Lower in the SSC group (132±12 bpm) vs. SSC + maternal singing group (145±15 bpm, p=.04)			

			singing, and ended with 10 min of SSC alone. Maternal singing was guided by a music therapist who instructed mothers to sing songs in a soothing tone that was accompanied by the music therapist via guitar. Music was adapted to the infant's reaction.			**Infant RR (mean±SD):** Lower for SSC group (51±7 per min) vs. SSC + maternal singing group (63±9 per min, p=.04)<br><br>**Infant SpO_2 (mean±SD):** Higher for SSC group (95±2%) vs. SSC + maternal singing group (91±6%, p=.04)<br><br>**Infant behavioral state (median [IQR]):** Lower for SSC group (1, IQR 1-3) vs. SSC + maternal singing group (3, IQR 2-5; p=.04)<br><br>**Maternal HR (mean±SD):** No significant difference between SSC (65±9 bpm) and SSC + maternal singing (79±15 bpm, p=.07)<br><br>**Maternal SpO_2 (mean±SD):** No difference between SSC (98±1%) and SSC + maternal singing (96±2%, p=.15)<br><br>**Maternal anxiety (mean±SD):** Lower for SSC (31.5±7.3) vs. SSC + maternal singing (39.1±10.4, p=.04)
**Fontana, 2020, Italy	57	Randomized Controlled Trial	2 groups: early intervention vs control (standard care)<br><br>Early intervention: Kangaroo Care, Parent training started one week after birth if clinically stable and 3 weeks later, massage started. Visual intervention started at 34 weeks. In the EI group the massage therapy was started by parents at [mean (SD)] 32.1 (1.1) weeks of GA and carried out for 9.5 (2.1) times a week. Visual interaction was proposed starting from	GA: 25-29 weeks<br><br>Mean GA: 28.4 (EI group)<br><br>27.8 (controls)<br><br>Intervention started 3 weeks following birth mean 32.1 weeks GA	Visual function (9-items measuring ocular spontaneous motility, ability to fix and follow a target, track a colored stimulus, stripes discrimination, and attention at distance) at term equivalent age	**All 9 items of visual function:** 59% (16/27) of the early intervention group achieved the highest score vs. 17% (5/30) of the controls (p=.001)<br><br>**Spontaneous ocular motility (mainly conjugated):** 96.3% (26/27) of early intervention vs. 70% (21/30) controls (p=.013)<br><br>**Ocular movements with target (mainly conjugated):** 85.2% (23/27) early intervention vs. 53.3% (16/30) controls (p=.012)<br><br>**Fixation (Stable, >3 s):** both groups achieved 100%<br><br>**Tracking – horizontal (complete):** both groups achieved 100%<br><br>**Tracking – vertical (complete):** 100% (27/27) early intervention vs. 96.7% (29/30) controls (p=1.0)<br><br>**Tracking – arc (complete):** 100% (27/27) early intervention vs. 80% (24/30) controls (p=.025)

			34.9 (0.7) weeks of GA and performed 6.2 (1.6) times a week.<br><br>Standard Care: kangaroo care, nesting, and minimal handling			**Tracking colored stimulus (present):** both groups achieved 100%<br><br>**Stripes discrimination (7-8 cards):** 77.8% (21/27) early intervention vs. 33.3 (10/30) controls (p=.001)<br><br>**Attention at distance (≥70 cm):** 74.1% (20/27) early intervention vs. 20% (6/30) controls (p<.001)
**Haslbeck 2020 and<br><br>Kehl 2020, Switzerland	82<br><br>32 parents	Randomized Controlled Trial<br><br>Sub-study	2 groups: creative music therapy (infant touch, observation, humming that transitioned to lullaby style tailored to breathing and state of infant) vs control (standard care)<br><br>Intervention: 20 min, 2 to 3 times per week, after morning feeding, at bedside or during skin-to-skin. Minimum of 8 sessions per infant. When tolerant to touch, session started with initial touch and transformed to therapeutic touch.	GA: < 32 weeks<br><br>Mean GA: 27.99±2.08<br><br>Started at chronological age ≥ 7 days of life	Haslbeck 2020: Brain structure and function;<br><br>Kehl 2020: Parental symptoms of anxiety, depression, stress, and parent-infant attach-ment	**Structural brain connectivity:** moderately affected by CMT<br><br>**Structural connectomic analysis:** there was increased integration in the posterior cingulate cortex only in the intervention group<br><br>Lagged resting-state MRI analysis showed lower thalamocortical processing delay, stronger functional networks, and higher functional integration in predominantly left prefrontal, supplementary motor, and inferior temporal brain regions in infants treated with CMT<br><br>**Anxiety:** no significant differences in parental anxiety levels at all time points (p>0.05)<br><br>**Depression:** no significant differences between groups, but within the music therapy group there was a significant reduction in depressive symptoms from second to third time period (p=0.025)<br><br>**Stress**: no significant differences between groups regarding stress measures<br><br>**Parent-infant attachment:** no significant difference between groups at first time period; at second time period Music therapy group showed increased parent-infant attachment compared to controls (i.e. shorter Self-Baby distance, p=0.026). but at third time period there was no significant difference between groups

**Jaywant, 2020, India	60	Randomized controlled trial	PIOMI alone: Eight steps of PIOMI (C-stretch, lip roll, lip curl, gum massage, cheek massage or lateral border of the tongue, mid-blade of the tongue stimulation, eliciting suck, nonnutritive sucking) for 10 minutes twice a day;<br><br>PIOMI + massage: Graded massage on head, neck, back, swaddling in prone and supine followed by graded massage on all extremities (30 seconds for each step) for 5 minutes, followed by PIOMI as described above for 5 minutes twice a day.<br><br>Interventions continued in both groups until full oral feeds were achieved.	Included GA 32-36 weeks;<br><br>EGA 32.64±1.9 for PIOMI alone and 32.78±2.21 for PIOMI + massage; intervention started at PMA 33 weeks in both groups	Ages and Stages Social Emotional Questionnaire-2 (at 2 and 6 months; higher scores indicate more delay)	**Ages and Stages Questionnaire – 2 months (mean ± SD):** Lower for PIOMI + massage (18.4±4.5) vs. PIOMI alone (30.4±7.4, p<.00001)<br><br>**Ages and Stages Questionnaire – 6 months (mean ± SD):** Lower for PIOMI + massage (28.2±8.14) vs. 46.2±11.57, p<.00001)<br><br>Data reported graphically showed the PIOMI only group exhibited more difficulty in communication and social interaction.
*Küçük Alemdar 2020, Turkey	136	Randomized Controlled Trial	4 groups: recorded maternal voice vs odor of breast milk vs incubator cover vs standard care<br><br>Maternal voice: mothers were recorded expressing thoughts and feelings, recording then played at 45dB inside	Mean GA: 30.26±0.69 (odor group)<br><br>30.06±0.63 (voice group)<br><br>30.22±0.66 (incubator)<br><br>30.25±0.5 (control)	Weight, height, head circumference, SO2, heart rate, respiratory rate, transition to oral feeding	No statistically significant difference in weight, height, head circumference, heart rate, or respiratory rate<br><br>**SO2:** mean SO2 values were statistically higher in the IC group than the other groups<br><br>**Oral feeding:** the briefest duration of transition to total oral feeding was seen in the odor group

			incubator while awake, 3 times a day for 30 min<br><br>Breastmilk odor: 5 ml of milk poured into sterile sponge and positioned 5 cm away from infant. Exposed once a day for 3 h, until infant transitioned to oral feed<br><br>Incubator: cloth was draped over incubator until transitioned to oral feeding			
*Meder, 2020, Hungary	31	Single group repeated measures	Single group of infants received four phases of measurement. Baseline measurement for 30 min in the incubator was followed by 30 min maternal SSC. This was followed by 20 min SSC + live maternal singing and guitar music, followed by a final phase with 30 min SSC alone. The intervention was performed in the afternoon at least 20 min after feeding.	Included GA 23-36 weeks; median EGA 30 (IQR 26, 32);<br><br>infants had to be at least 5 days old to be included	rSO_2, HR, SpO_2, cFTOE	**rSO_2 (mean±SD, %):** Increased from baseline (76.87 ± 2.97) to final SSC (78.00 ± 3.04, p=.02); no significant difference in the coefficient of variation during intervention phases compared to baseline (p=.052)<br><br>**SpO_2 (mean±SD, %):** No difference from baseline (93.07 ± 4.11) to final SSC (94.10 ± 3.77, p=.17); the coefficient of variation was lower during intervention phases compared to baseline (p<.001)<br><br>**HR (mean±SD, bpm):** No difference from baseline (154.3 ± 16.7) to final SSC (155.6 ± 13.7, p=.59); no difference in the coefficient of variation during in-tervention phases compared to baseline (p=.49)<br><br>**cFTOE (mean±SD):** No difference from baseline (0.17 ± 0.04) to final SSC (0.17 ± 0.03, p=.72)
**Pineda, 2020, USA	80	Quasi-experimental	SENSE: parent educa-tional materials and tar-geted minimum amounts of sensory in-terventions by term equivalent age. Interventions and targeted minimums included: tactile (gentle human touch, SSC, or	Included GA $\leq$32 weeks; EGA 27.6±2.6 for controls and 28.6±2.4 for SENSE group; infants were enrolled in the first week of life	Maternal outcomes prior to infant dis-charge (37-41 weeks PMA): Parental Stress Scale, State Trait Anxi-ety Inventory, Edin-burgh Postnatal De-pression Scale, Modi-fied Perinatal PTSD questionnaire,	Maternal Outcomes (all values mean ± SD)<br><br>**Parental Stress Scale:** No difference between historical controls (26.7±5.4) and SENSE (26.3±6.2, p=.73)<br><br>**Edinburgh Postnatal Depression Scale:** No difference between historical controls (7.08±4.2) and SENSE (8.5±5.9, p=.27)

			massage) for 3 hr; auditory (language or music) for 3 hr; visual (dim and cycled light with human interactions); vestibular (rocking) for 7 min; kinesthetic (opportunities for free movement for 2 min prior to diaper changes); and olfactory exposures (scent cloth and close maternal contact). When parents were not able to complete SENSE interventions, OT graduate students read to the infant or provided gentle human touch.<br><br>Historical controls: standard of care, which included promotion of SSC holding and other developmental care at the discretion of the parents and NICU care team		Parental Role Alterations subscale from the Parental Stressor Scale: NICU, Maternal Confidence Questionnaire, and Infant Care Questionnaire. Infant outcomes at term equivalent age (37-41 weeks): NICU Network Neurobehavioral Scale, Hammersmith Neonatal Neurological Assessment.	**Anxiety (State Trait Anxiety Scale Inventory-State):** no difference between historical controls (30.4±7.9) and SENSE (29.5±8.7, p=.67)<br><br>**Anxiety (State Trait Anxiety Scale Inventory-Trait):** no difference between historical controls (30.1±8.5) and SENSE (28.0±8.6, p=.36)<br><br>**Parental Stress Scale-NICU:** no difference between historical controls (2.8±0.8) and SENSE (2.8±1.13, p=.80)<br><br>**Maternal Confidence Questionnaire:** lower for historical controls (41.4±7.6) vs. SENSE (49.5±6.1, p=.01 after controlling for maternal and infant factors)<br><br>**Infant Care Questionnaire – mom and baby:** no difference between historical controls (4.3±0.4) and SENSE (4.5±0.4, p=.24 after controlling for maternal and infant factors)<br><br>**Infant Care Questionnaire – emotionality:** no difference between historical controls (1.3±0.9) and SENSE (4.6±0.5, p=.18)<br><br>**Infant Care Questionnaire – responsiveness:** no difference between historical controls (4.0±0.5) vs. SENSE (4.0±0.5, p=.84)<br><br>**Modified Perinatal PTSD:** no difference between historical controls (8.25±7.6) vs. SENSE (6.23±7.6, p=.33)<br><br><u>Infant Outcomes (all values mean ± SD)</u><br><br>**NICU Network Neurobehavioral Scale:** no difference in 11 of 12 summary scores; historical controls exhibited more asymmetry (2.9±1.8) compared to SENSE group (2.0±1.3, p=.02 after adjusting for maternal and infant factors)

						**Hammersmith Neonatal Neurological Evaluation:** lower for historical controls (15.3±4.2) vs. SENSE group (20.1±4.6, $p<0.001$)
**Abbreviations:** BMD, bone mineral density; BP, blood pressure; bpm, beats per minute; cFTOE, cerebral fractional tissue oxygen extraction; EGA, estimated gestational age; GA, gestational age; HR, heart rate; IQR, interquartile range; IVH, intraventricular hemorrhage; KC, kangaroo care; MT, music therapy; MTT, maternal therapeutic touch; MVS, maternal voice stimulus; NICU, neonatal intensive care unit; PIOMI, Premature Infant Oral Motor Intervention; PMA, postmenstrual age; PVL, periventricular leukomalacia; RR, respiratory rate; rSO2, regional cerebral oxygenation; SD, standard deviation; SDF, supported diagonal flexion; SENSE, Supporting and Enhancing NICU Sensory Experiences; SpO_2, oxygen saturation; SSC, skin-to-skin contact; VC, visual contact.						

*Articles that solely have outcomes of physiology, bone health, gastrointestinal function, growth, and length of stay are reported here, but were not used for refinement of the SENSE program due to a departure from the main outcomes of interest.

**Articles that have at least one measure related to the main outcomes of interest, infant neurobehavior or neurodevelopmental outcome.
