# Supplementary material for: NICU sensory experiences associated with positive outcomes: an integrative review of evidence from 2015–2020
Source: J Perinatol. 2023 Apr 7;43(7):837–48. doi: 10.1038/s41372-023-01655-y (PMC10325947; doi:10.1038/s41372-023-01655-y)
Supplement: Supplementary file 2 — Appendix B [file 41372_2023_1655_MOESM2_ESM.docx]

**Appendix B**

1. Quality assessment of included studies**.**

|  | **Selection Bias** | | | **Performance Bias** | | | **Attrition Bias** | | | **Detection Bias** | | | | **Other bias and comments** |
| --- | --- | --- | --- | --- | --- | --- | --- | --- | --- | --- | --- | --- | --- | --- |
| **Study, Year** | A1. Randomization | A2. Allocation concealment | A3. Comparable at baseline | B1. Same care | B2. Blinded participants | B3. Blinded healthcare workers | C1. Equal follow-up time | C2. Comparable for treatment completion | C3. Comparable for outcome data | D1. Appropriate length of follow-up | D2. Precise definition of outcome | D3. Valid and reliable outcome | D4. Investigators blind to exposure | \| **Key** \| \| \| --- \| --- \| \| Yes \|  \| \| Unclear \|  \| \| No \|  \| \| Unable to assess \|  \| |
| **Tactile** | | | | | | | | | | | | | | |
| Cho 2016 |  |  |  |  |  |  |  |  |  |  |  |  |  | No mention of randomization or allocation concealment. No blinding. |
| Coskun 2019 |  |  |  |  |  |  |  |  |  |  |  |  |  | Minimal assessment of baseline factors. Possible differences in some baseline factors, but not statistically significant. |
| Diniz 2019 |  |  |  |  |  |  |  |  |  |  |  |  |  | Minimal assessment of baseline factors. |
| Dongre 2020 |  |  |  |  |  |  |  |  |  |  |  |  |  | Single group studies cannot be assessed for all factors but are considered lower quality designs. |
| El-Farrash 2019 |  |  |  |  |  |  |  |  |  |  |  |  |  | Participants received varying doses of the intervention. |
| Forde 2020 |  |  |  |  |  |  |  |  |  |  |  |  |  | Imbalance in ethnicity at study start and non-significant imbalance in gender. |
| Mehler 2020  Hucklenbruch-Rother 2020 |  |  |  |  |  |  |  |  |  |  |  |  |  | Some outcomes were self-reported and unable to be blinded. |
|  |  |  |  |  |  |  |  |  |  |  |  |  |  |  |
| Kurt 2020 |  |  |  |  |  |  |  |  |  |  |  |  |  | Minimal assessment of baseline factors. Unclear if participants received similar care as groups were studies at two different hospitals. Statistician was blinded, but unclear blinding of data collectors. |
| Özdel 2018 |  |  |  |  |  |  |  |  |  |  |  |  |  | Unclear if length of follow-up is too short to adequately assess outcome (<24 hours). |
| Shattnawi 2019 |  |  |  |  |  |  |  |  |  |  |  |  |  | Minimal assessment of baseline factors. Participants received varying doses of the intervention. |
| Sehgal 2020 |  |  |  |  |  |  |  |  |  |  |  |  |  | Single group studies cannot be assessed for all factors but are considered lower quality designs. |
| Vittner 2018 |  |  |  |  |  |  |  |  |  |  |  |  |  | Did not compare baseline factors of randomized groups. |
| **Auditory** | | | | | | | | | | | | | | |
| Jabraeili 2016 |  |  |  |  |  |  |  |  |  |  |  |  |  | Minimal baseline assessment. Described as double blind but does not specify who was blinded. Different loss to follow-up rates across intervention groups. |
| Lejeune 2019a  Lordier 2019a  Lordier 2019b  Sa de Almeida 2020 |  |  |  |  |  |  |  |  |  |  |  |  |  | Due to loss to follow-up in some studies, the profiles of patients differed slightly across publications. In some cases, 50% of the population was excluded or lost to follow-up. Unclear if all outcome assessment was blinded. |
|  |  |  |  |  |  |  |  |  |  |  |  |  |  |  |
| Lejeune 2019b |  |  |  |  |  |  |  |  |  |  |  |  |  | Some imbalances at baseline within voice condition (+5 vs. +15 dBA) and between silence and voice groups. Loss of 20 infants in the voice condition (unclear +5/+15 dBA) at the study start. |
| Nöcker-Ribaupierre 2015 |  |  |  |  |  |  |  |  |  |  |  |  |  | Some outcomes had long follow-up periods with unclear losses. Unclear validity/reliability of the outcomes obtained from maternal diaries. Not all outcome assessment was blinded. |
|  |  |  |  |  |  |  |  |  |  |  |  |  |  |  |
| Ranger 2018 |  |  |  |  |  |  |  |  |  |  |  |  |  | Did not compare at baseline. Unclear how randomization occurred across settings or whether care was similar across settings. Some calculated outcomes had unclear clinical relevance. Some data from the second crossover period were excluded due to carry over effects. |
| Shafiei 2020 |  |  |  |  |  |  |  |  |  |  |  |  |  | Randomized groups not compared at baseline. |
| **Vision** | | | | | | | | | | | | | | |
| Brandon 2017 |  |  |  |  |  |  |  |  |  |  |  |  |  | Assessed multiple factors at baseline, so some differences may be due to chance. Unclear if all outcome assessment was blinded. |
|  |  |  |  |  |  |  |  |  |  |  |  |  |  |  |
| Kaneshi 2016 |  |  |  |  |  |  |  |  |  |  |  |  |  | Minimal assessment of factors at baseline. |
| Lebel 2017 |  |  |  |  |  |  |  |  |  |  |  |  |  | Follow-up period was minimal (24 hours). |
| Tandircioglu 2019 |  |  |  |  |  |  |  |  |  |  |  |  |  | Possible imbalances at baseline but sample size was small. |
| **Kinesthetic** | | | | | | | | | | | | | | |
| Litmanovitz 2016 |  |  |  |  |  |  |  |  |  |  |  |  |  | Randomization and allocation concealment were unclear; described as “opaque envelope randomization.” |
| Sezer Efe 2020 |  |  |  |  |  |  |  |  |  |  |  |  |  | Weak baseline assessment. Attending physicians were blinded but not other healthcare worker groups. Significant losses to follow-up in both groups (50% exercise group and 40% controls). |
|  |  |  |  |  |  |  |  |  |  |  |  |  |  |  |
| Ustad 2016, Fjørtoft 2017, Øberg 2020 |  |  |  |  |  |  |  |  |  |  |  |  |  | Some imbalances at baseline but tested more factors than most studies. Larger losses in the intervention group after allocation to the intervention. |
| **Olfactory/Gustatory** | | | | | | | | | | | | | | |
| Russell 2015 |  |  |  |  |  |  |  |  |  |  |  |  |  | Only a small number of factors were assessed across group at baseline (EGA, Apgar, day of life). |
| **Multimodal** | | | | | | | | | | | | | | |
| Acosta 2018 |  |  |  |  |  |  |  |  |  |  |  |  |  | Minimal assessment of factors at baseline. Unclear blinding of care givers and data collectors. |
| Álvarez 2019 |  |  |  |  |  |  |  |  |  |  |  |  |  | Minimal assessment of factors at baseline. Some differences in factors such as sex but were not significantly different. |
| Baby 2015 |  |  |  |  |  |  |  |  |  |  |  |  |  | Vague description of statistical methods, unclear reporting of paired-t analysis. |
| Banisadi 2019 |  |  |  |  |  |  |  |  |  |  |  |  |  | Single group studies cannot be assessed for all factors but are considered lower quality designs. Unclear how many infants completed treatment versus missing outcome data. |
| Buil 2019a  Buil 2019b |  |  |  |  |  |  |  |  |  |  |  |  |  | Possible differences in some factors at baseline but were not statistically different. |
| Carvalho 2019 |  |  |  |  |  |  |  |  |  |  |  |  |  | Did not compare group characteristics at baseline. |
| Choi 2016 |  |  |  |  |  |  |  |  |  |  |  |  |  | Minimal baseline assessment. Unclear how many participants were loss to follow-up in each group. |
| Detmer 2019 |  |  |  |  |  |  |  |  |  |  |  |  |  | Minimal baseline assessment. Unclear how many participants were loss to follow-up in each group. |
| Efendi 2018 |  |  |  |  |  |  |  |  |  |  |  |  |  | Possible differences in some baseline factors but were not statistically different. |
| El-Farrash 2019 |  |  |  |  |  |  |  |  |  |  |  |  |  | Blinding of neonatologists, but unclear if all caretakers were blinded to treatment allocation. |
|  |  |  |  |  |  |  |  |  |  |  |  |  |  |  |
| Elmoneim 2021 |  |  |  |  |  |  |  |  |  |  |  |  |  | Some non-significant imbalances at baseline (sex, O_2_). Only DXA scan readers were blinded to group allocation. |
|  |  |  |  |  |  |  |  |  |  |  |  |  |  |  |
| Epstein 2020 |  |  |  |  |  |  |  |  |  |  |  |  |  | Only compared PMA of randomized groups at baseline. |
| Ettenberger 2016 |  |  |  |  |  |  |  |  |  |  |  |  |  | Minimal baseline assessment. Possible differences in some baseline factors, but no statistically significant difference. |
| Fontana 2020 |  |  |  |  |  |  |  |  |  |  |  |  |  | Baseline factors were not statistically compared though most appeared similar. |
| Haslbeck 2020  Kehl 2020 |  |  |  |  |  |  |  |  |  |  |  |  |  | Study groups in the Kehl 2020 sub-study were not balanced at baseline. Missing outcome data for large numbers in Haslbeck 2020. |
|  |  |  |  |  |  |  |  |  |  |  |  |  |  |  |
| Jaywant 2020 |  |  |  |  |  |  |  |  |  |  |  |  |  | Minimal baseline assessment. |
| Konar 2019 |  |  |  |  |  |  |  |  |  |  |  |  |  | Minimal baseline assessment. |
| Küçük Alemdar 2020 |  |  |  |  |  |  |  |  |  |  |  |  |  | Minimal baseline assessment. |
| Meder 2020 |  |  |  |  |  |  |  |  |  |  |  |  |  | Single group studies cannot be assessed for all factors but are considered lower quality designs. |
| Pineda 2020 |  |  |  |  |  |  |  |  |  |  |  |  |  | Intervention and control periods separated by 4 years. |
| Taheri 2018 |  |  |  |  |  |  |  |  |  |  |  |  |  | Possible differences in some baseline factors, but not statistically significant. |
| Vahdati 2017 |  |  |  |  |  |  |  |  |  |  |  |  |  | Unclear how randomization process was conducted over the entire study period. Minimal assessment of baseline factors. Possible differences in some baseline factors, but not statistically significant. |
| **Note:** Cells with two colors in a single cell indicates that some study characteristics met one condition, while other characteristic met another condition.  *Assessed for quality using a tool adapted from: National Institute for Health and Care Excellence (NICE). Appendix C: Methodology checklist: randomized controlled trials. *The Guidelines Manual* 2012: <http://publications.nice.org.uk/the-guidelines-manual-appendices-bi-pmg6b/appendix-c-methodology-checklist-randomised-controlled-trials>. | | | | | | | | | | | | | | |
