# Supplementary material for: NICU sensory experiences associated with positive outcomes: an integrative review of evidence from 2015–2020
Source: J Perinatol. 2023 Apr 7;43(7):837–48. doi: 10.1038/s41372-023-01655-y (PMC10325947; doi:10.1038/s41372-023-01655-y)
Supplement: Supplementary file 3 — Appendix C [file 41372_2023_1655_MOESM3_ESM.docx]

Appendix C. Different sensory interventions studied across postmenstrual age (PMA) from 1995-2020 (inclusive of previous and current integrative reviews). The number in each box indicates how many studies were conducted with at least some of the sample receiving interventions at that particular PMA.

|  | Studies with one or more positive outcomes | c | Most of the studies had one or more positive outcomes; some studies had statistically significant findings that were questionable as to whether they were positive or negative |
| --- | --- | --- | --- |
|  | Studies with one or more statistically significant findings that were questionable as to whether they were positive or negative |  | Most of the studies had one or more positive outcomes; some studies had statistically significant findings that were questionable as to whether they were positive or negative |
|  | Studies were conducted, but none of the findings reached statistical significance |  | Most of the studies had one or more positive outcomes; some studies did not have findings that reached statistical significance |
|  |  | c | Most of the studies had one or more positive outcomes; some studies had statistically significant findings that were questionable as to whether they were positive or negative, and some studies had one or more adverse outcomes |

|  | Postmenstrual age (weeks) | | | | | | | | | | | | | | | | | |
| --- | --- | --- | --- | --- | --- | --- | --- | --- | --- | --- | --- | --- | --- | --- | --- | --- | --- | --- |
|  | 23 | 24 | 25 | 26 | 27 | 28 | 29 | 30 | 31 | 32 | 33 | 34 | 35 | 36 | 37 | 38 | 39 | 40 |
| **Tactile Interventions** | | | | | | | | | | | | | | | | | | |
| **Gentle human touch |  |  |  |  | 1 | 1 | 2 | 2 | 2 | 3 | 3 | 3 | 2 | 1 | 1 |  |  |  |
| **Skin-to-skin/Kangaroo care |  | 3 | 6 | 8 | 9 | 12 | 14 | 17 | 20 | 26 | 23 | 24 | 22 | 19 | 12 | 8 | 6 | 5 |
| **Auditory Interventions** | | | | | | | | | | | | | | | | | | |
| **Live/recorded music |  |  | 1 | 1 | 1 | 2 | 2 | 3 | 4 | 6 | 7 | 7 | 4 | 4 | 3 | 2 | 2 | 2 |
| **Voice/human sounds |  |  | 1 | 1 | 2 | 4 | 6 | 7 | 7 | 7 | 7 | 6 | 4 | 2 | 2 | 2 | 2 | 1 |
| **Visual Interventions** | | | | | | | | | | | | | | | | | | |
| Darkness |  |  |  |  |  |  |  | 2 | 2 | 2 | 1 | 1 | 1 |  |  |  |  |  |
| Red light |  |  |  |  |  |  |  |  |  |  |  |  | 1 | 1 | 1 | 1 | 1 |  |
| Cycled light | 1 | 1 | 1 | 1 | 1 | 3 | 4 | 5 | 5 | 4 | 3 | 3 | 3 | 2 | 1 | 1 | 1 | 1 |
| **Kinesthetic Interventions** | | | | | | | | | | | | | | | | | | |
| Physical therapy |  |  |  |  |  |  |  | 1 | 1 | 1 | 2 | 2 | 1 | 1 | 1 |  |  |  |
| Passive range of motion |  |  |  |  |  | 1 | 1 | 2 | 2 | 2 | 1 | 1 |  |  |  |  |  |  |
| **Guided movement and position changes |  |  |  |  |  |  |  |  |  |  |  | 1 | 1 | 1 |  |  |  |  |
| **Gustatory/Olfactory Interventions** | | | | | | | | | | | | | | | | | | |
| Maternal scent/breast milk |  |  |  |  |  |  |  | 2 | 4 | 6 | 5 | 5 | 3 | 3 | 2 | 2 |  |  |
| Colostrum |  | 1 | 1 | 1 | 1 |  |  |  |  |  |  |  |  |  |  |  |  |  |
| **Multimodal Interventions** | | | | | | | | | | | | | | | | | | |
| **Supporting and Enhancing NICU Sensory Experience (SENSE) | 1 | 1 | 1 | 1 | 1 | 1 | 1 | 1 | 1 | 1 | 1 | 1 | 1 | 1 | 1 | 1 | 1 | 1 |
| **Fontana multisensory program |  |  |  | 1 | 1 | 1 | 1 | 1 | 1 | 1 | 1 | 1 | 1 | 1 | 1 | 1 | 1 | 1 |
| **Skin-to-skin care + auditory |  |  | 1 | 1 | 1 | 1 | 3 | 4 | 5 | 7 | 7 | 7 | 7 | 7 | 6 | 5 | 3 | 3 |
| **Massage (with a kinesthetic component) |  |  |  |  |  | 2 | 4 | 8 | 11 | 14 | 18 | 22 | 21 | 17 | 10 | 8 | 7 | 7 |
| **Massage + olfactory |  |  |  |  |  |  |  | 1 | 1 | 1 | 1 | 1 | 1 | 1 | 1 | 1 | 1 | 1 |
| **Massage + Premature Infant Oral Motor Intervention |  |  |  |  |  |  |  |  |  | 1 | 1 | 1 | 1 | 1 |  |  |  |  |
| **Therapeutic static touch + auditory |  | 2 | 2 | 2 | 2 | 2 | 3 | 3 | 3 | 3 | 3 | 3 | 3 | 3 | 3 | 3 | 3 | 3 |
| Skin-to-skin + range of motion |  |  |  |  |  |  | 1 | 1 | 1 | 1 | 1 | 1 | 1 | 1 | 1 |  |  |  |
| **Audo, Tactile, Visual, Vestibular Intervention (ATVV) |  |  |  |  |  |  |  |  |  | 2 | 8 | 7 | 7 | 7 | 6 | 4 | 4 | 4 |
| **Family nurture |  |  |  |  |  |  |  |  | 2 | 2 | 2 | 2 | 2 | 2 | 2 | 2 | 2 | 2 |
| H-Hope |  |  |  |  |  |  |  |  |  | 2 | 2 | 2 | 2 | 2 | 2 |  |  |  |

**Interventions that have at least one measure related to the main outcomes of interest, infant neurobehavior or neurodevelopmental outcome.
